# Conversion of the CG specific M.MpeI DNA methyltransferase into an enzyme predominantly methylating CCA and CCC sites

**DOI:** 10.1093/nar/gkad1217

**Published:** 2024-01-02

**Authors:** Pál Albert, Bence Varga, Györgyi Ferenc, Antal Kiss

**Affiliations:** Laboratory of DNA-Protein Interactions, Institute of Biochemistry, HUN-REN Biological Research Centre, 6726 Szeged, Hungary; Doctoral School of Biology, Faculty of Science and Informatics, University of Szeged, 6726 Szeged, Hungary; Laboratory of DNA-Protein Interactions, Institute of Biochemistry, HUN-REN Biological Research Centre, 6726 Szeged, Hungary; Doctoral School of Biology, Faculty of Science and Informatics, University of Szeged, 6726 Szeged, Hungary; Nucleic Acid Synthesis Laboratory, Institute of Plant Biology, HUN-REN Biological Research Centre, 6726 Szeged, Hungary; Nucleic Acid Synthesis Laboratory, Institute of Plant Biology, HUN-REN Biological Research Centre, 6726 Szeged, Hungary; Laboratory of DNA-Protein Interactions, Institute of Biochemistry, HUN-REN Biological Research Centre, 6726 Szeged, Hungary

## Abstract

We used structure guided mutagenesis and directed enzyme evolution to alter the specificity of the CG specific bacterial DNA (cytosine-5) methyltransferase M.MpeI. Methylation specificity of the M.MpeI variants was characterized by digestions with methylation sensitive restriction enzymes and by measuring incorporation of tritiated methyl groups into double-stranded oligonucleotides containing single CC, CG, CA or CT sites. Site specific mutagenesis steps designed to disrupt the specific contacts between the enzyme and the non-substrate base pair of the target sequence (5′-CG/5′-CG) yielded M.MpeI variants with varying levels of CG specific and increasing levels of CA and CC specific MTase activity. Subsequent random mutagenesis of the target recognizing domain coupled with selection for non-CG specific methylation yielded a variant, which predominantly methylates CC dinucleotides, has very low activity on CG and CA sites, and no activity on CT sites. This M.MpeI variant contains a one amino acid deletion (ΔA323) and three substitutions (N324G, R326G and E305N) in the target recognition domain. The mutant enzyme has very strong preference for A and C in the 3′ flanking position making it a CCA and CCC specific DNA methyltransferase.

## Graphical Abstract

**Graphical Abstract:**
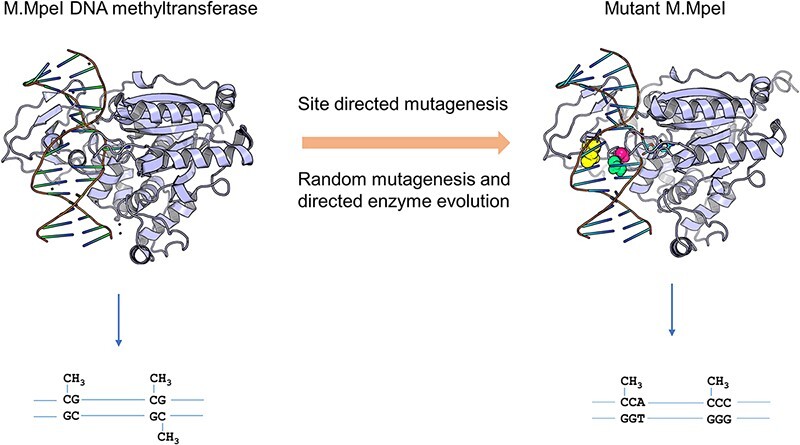

## Introduction

In the genome of many organisms some cytosines or adenines are in methylated form (C5-methylcytosine, N4-methylcytosine and N6-methyladenine). N6-adenine methylation is widespread in prokaryotes and can be found in the genome of some lower eukaryotes (1). N4-cytosine methylation was thought to occur only in prokaryotes, but a recent study identified N4-cytosine methylation in an invertebrate eukaryote (2). C5-cytosine methylation is common in prokaryotes and eukaryotes as well (3). In prokaryotes DNA methylation plays roles mainly in restriction-modification, whereas in higher eukaryotes including humans it is part of epigenetic gene regulation. The methylated bases are created by DNA methyltransferases (MTases), which transfer a methyl group from *S*-adenosyl-methionine (SAM) onto cytosines or adenines in specific nucleotide sequences (3).

Bacterial C5-MTases share structural similarity with the catalytic domain of eukaryotic C5-MTases: they contain ten conserved sequence motifs and a target recognizing domain (TRD), the latter being mainly responsible for recognition of the substrate site (4–8). The catalytic mechanism involves flipping the target cytosine out of the double helix, and formation of a transient covalent bond between the enzyme's catalytic nucleophile and carbon 6 of the target cytosine (9,10).

In mammalian genomes C5-methylcytosines (^m5^C) are found mainly in CG dinucleotides. However, in pluripotent and brain cells C5-methylcytosines occur, besides the predominant CG context, also in CH dinucleotides, where H stands for A, C or T. The biological role of non-CG specific DNA methylation is unclear (11,12).

The bacterial C5-MTases M.SssI (13) and M.MpeI (14,15) share the specificity of eukaryotic MTases (CG), thus have the potential to become research tools for studying eukaryotic DNA methylation. M.SssI has already found applications in targeted DNA methylation strategies (16–20). To our knowledge no C5-MTases with CH, CA, CC or CT specificities are known to exist (21).

In this work we tried to convert the CG specific MTase M.MpeI into an enzyme that has acquired the capacity to methylate any one or a combination of CA, CC or CT sites, but has lost its original CG specific MTase activity. We chose M.MpeI because it is structurally better characterized than M.SssI: for M.MpeI we have an X-ray structure of the specific recognition complex formed by the enzyme and cognate DNA (15), whereas for M.SssI only a computationally generated model is available (22). Moreover, in our hands M.MpeI was more active, more robust and easier to work with than M.SssI (23). We used a combination of rational and random mutagenesis guided by the X-ray structure of the specific M.MpeI-DNA recognition complex (15) to change the substrate preference of M.MpeI. The starting goal was to perturb recognition of the 3′-half of the target sequence (5′-CG/5′-CG). In this context it is important to note that although the M.MpeI target sequence is a palindrome, the two strands are methylated in two independent binding events, thus in the enzyme-DNA complex the DNA is asymmetric with a defined 5′ to 3′ orientation.

Multiple steps of site directed mutagenesis designed to disrupt the specific contacts between the enzyme and DNA yielded M.MpeI variants with varying levels of the original CG specific and increasing levels of CA and CC specific MTase activity. A further shift in methylation specificity was achieved by random mutagenesis and selection for non-CG specific methylation activity. The best mutant mainly methylates CC dinucleotides in CCA and CCC contexts. It has very low activity on CA and CG sites and does not methylate CT sites.

## Materials and methods

### Strains, media and growth conditions

Plasmids were constructed in *Escherichia coli* DH10B (24). Methylation specificity of MTase variants was tested in *Escherichia coli* ScarabXpress T7 *lac* (Scarab Genomics) or in *E. coli* ER1821 (25). Bacteria were grown in LB or TB medium (26) supplemented with 50 μg/ml kanamycin (Kn) at 30 or 37°C.

### Plasmids

Plasmids used in this work are listed in Supplementary Table S1. The plasmid pET28-MMpeI (Kn^R^) originally named pET-28a::Mmpe (15) encodes a C-terminally His-tagged variant of wild-type M.MpeI. In pET28-MMpeI the M.MpeI gene is transcribed from the T7 promoter, and expression can be induced with isopropyl β-d-1-thiogalactopyranoside (IPTG) (15). Some mutant M.MpeI genes were cloned in the plasmid vector pOK-BAD (Kn^R^) (27). In the latter plasmids the M.MpeI gene is transcribed from the arabinose inducible *E. coli araBAD* promoter.

Plasmids carrying site directed mutations in the M.MpeI gene were constructed by inverse PCR (28) using mutagenic oligonucleotide primers listed in Supplementary Tables S2 and S3.

To transfer the gene variant encoding M.MpeI(ΔA323) from pET28-MMpeI(ΔA323) into pOK-BAD (27), the MTase coding sequence was PCR amplified using the primers AK463 and AK739, then the PCR product was digested with NcoI and HindIII, and cloned between the NcoI and HindIII sites of the expression plasmid vector pOK-BAD to obtain pOB-MMpeI(ΔA323).

Variants of the plasmid pOB-MMpeI(ΔA323+N324G+R326G+E305N) with a second BamHI site were constructed by cloning the AK946-AK947 or AK948-AK949 double stranded oligonucleotides (Supplementary Table S2) between the Eco31I and Psp1406I sites of the plasmid. The new plasmids were named to indicate the top strand of the oligonucleotide duplex, such as pOB-MMpeI(ΔA323+N324G+R326G+E305N)-AK946 (Supplementary Table S1).

### Oligonucleotides

Deoxyoligonucleotides (Supplementary Table S2) were synthesized by standard beta-cyanoethyl phosphoramidite chemistry on a nominal scale of 0.2 μmol in a DNA/RNA/LNA H-16 synthesizer (K&A Laborgeräte). The reagents for oligonucleotide synthesis were from Link Technologies, Sigma Aldrich, ChemGenes and Molar Chemicals Kft.

### DNA techniques

DNA cloning, PCR reactions and agarose gel electrophoresis of DNA fragments were done by standard methods (26). DNA fragments separated by agarose gel electrophoresis were visualized by staining with ECO Safe Nucleic Acid Staining Solution (Pacific Image Electronics) or SYBR Safe DNA Gel Stain (Invitrogen). GeneRuler 1 kb DNA Ladder (Thermo Scientific) was used as size marker. Enzymes were purchased from Thermo Scientific and New England Biolabs.

Site directed mutagenesis was performed by inverse PCR (28) using mutagenic oligonucleotides listed in Supplementary Table S3. Random mutagenesis was carried out by error prone PCR (29).

Nucleotide sequence of relevant parts of new plasmids was determined by automated DNA sequencing.

### Selection of altered specificity M.MpeI clones

Plasmids expressing M.MpeI mutants with non-CG specific MTase activity were selected by a method based on self-methylation of the plasmid by the encoded mutant MTase and the acquired resistance to restriction digestion (8).

A segment of the M.MpeI(ΔA323+N324G+E305A) gene extending from the beginning of the variable region to the 3′-end of the MTase coding sequence was mutagenized by error prone PCR. The reaction contained 1 ng pET28-MMpeI(ΔA323+N324G+E305A) plasmid DNA, 0.2 μM AK714 and AK715 primers, 1× Taq buffer, 9 mM MgCl_2_, 2 mM MnCl_2,_ 200 μM dNTPs, 160 μM dITP and 2 U Taq DNA polymerase in 50 μl. The AK714 and AK715 primers contained XbaI and XhoI sites, respectively, as 5′-extensions (Supplementary Table S2). XbaI cuts at the codon of Leu241, whereas the XhoI site overlaps the end of the MTase coding sequence.

The PCR steps were as follows: initial denaturation at 94°C for 3 min, 30 cycles of amplification (94°C for 30 s, 47°C for 30 s and 72°C for 2 min), final extension 72°C for 5 min. The PCR product was purified using GeneJET PCR purification kit (Thermo Scientific), and digested with XbaI and XhoI. The digested PCR product was cloned between the XbaI and XhoI sites of pOB-MMpeI(ΔA323). In pOB-MMpeI(ΔA323) the XbaI site in the M.MpeI gene is unique.

To select for altered specificity mutants, a culture containing the mutagenized plasmid library was grown in 100 ml LB plus kanamycin at 37 °C. At OD_600_ arabinose was added to 0.1% final concentration, and culturing was continued for 5 hours at 30 °C. Approximately 500 ng of the plasmid DNA purified from the culture was digested with 20 units of Eco47I in a final volume of 20 μl, and the digested DNA was used to transform *E. coli* ER1821 cells. Kn^R^ transformants were grown in the presence or absence of arabinose, and were used for plasmid preparation. Methylation status of the plasmid DNAs was analyzed by Eco47I, BsuRI and Bsh1236I digestions.

### Protein purification

To test MTase activity *in vitro*, *E. coli* ScarabXpress T7 *lac* cells containing the plasmid expressing the M.MpeI variant of interest were grown in TB medium (26), and MTase expression was induced by adding IPTG to the culture. Culturing was continued at 30°C for 5 hours. Cells were disrupted by sonication, and the His-tagged M.MpeI variant was purified by affinity chromatography using either His-Spin Protein Miniprep columns (Zymo Research) or conventional columns containing HIS-Select Nickel Affinity Gel (Sigma-Aldrich). Purity of the eluted samples was checked by SDS-polyacrylamide gel electrophoresis. The purified protein was concentrated using Pur-A-Lyzer Mini 12000 Dialysis Kit (Sigma-Aldrich) or conventional dialysis bags against a buffer containing 50 mM Tris–HCl (pH 7.5), 100 mM NaCl, 10 mM β-mercaptoethanol and 50% glycerol, and subsequently stored at −20°C. Concentration of the purified protein was determined by measuring UV absorption at 280 nm. In some cases protein concentration was estimated by SDS-polyacrylamide gel electrophoresis using protein samples of known concentration (Supplementary Figure S8).

### Characterization of DNA methyltransferase activity

*In vivo* activity and sequence specificity of the MTase variants were routinely tested by restriction protection assay. *E. coli* ScarabXpress T7 *lac* harboring pET28 based-plasmids expressing M.MpeI mutants was grown in LB supplemented with Kn at 37°C. MTase expression was induced at a cell density of OD_600_∼0.5 by adding 0.5 mM IPTG to the culture, then growth was continued for 5 h at 30°C. Plasmid DNA isolated from the cells was digested with restriction endonucleases sensitive to cytosine-C5 methylation in specific sequence contexts. MTase variants expressed from plasmids based on the vector pOK-BAD were tested in a same way, except that MTase expression was induced with 0.1% arabinose. Restriction protection of plasmids encoding M.MpeI variants was tested in at least two independent experiments.

To test MTase activity *in vitro*, the culture was grown in TB medium (26), and MTase expression was induced as described above. His-tagged M.MpeI variants were purified by affinity chromatography, and MTase activity of the purified enzyme variants was determined by measuring incorporation of tritiated methyl groups from *S*-adenosyl-l-[methyl-^3^H]methionine (PerkinElmer) into double-stranded oligonucleotide substrates. The assay was performed essentially as described previously (30,31). Standard MTase reactions contained 2 μM double-stranded oligonucleotide substrate DNA, 50 mM Tris–HCl pH 8.5, 50 mM NaCl, 10 mM EDTA, 5 mM DTT, 5 μM *S*-adenosyl-l-[methyl-^3^H]methionine, 0.1 mg/ml bovine serum albumin and purified M.MpeI (wild-type or mutant) in 30 μl end volume. The [methyl-^3^H]-SAM solution (specific activity: ∼130 Bq/pmol) was prepared by mixing *S*-adenosyl-l-[methyl-^3^H]methionine (555 Bq/pmol, PerkinElmer) and unlabeled SAM purchased from New England Biolabs. The oligonucleotide substrates were 23-mer duplexes, which had the same sequence except for the substrate CG/CA/CC/CT site and the flanking nucleotides preceding and following the substrate site (AK702 through AK709, AK921 through AK932 and AK967 through or AK994, Supplementary Tables S2 and S6). Because the non-canonical CA/CC/CT sites are asymmetric and can be methylated only on one strand, for reliable comparison the CG site in the canonical substrate was synthesized to contain C5-methylated CG on one strand (AK702-703, Supplementary Tables S2 and S6). The samples were incubated at 30°C for 30 min, then the reaction was stopped by adding 4 μl of 10% SDS, the samples were processed (30,31), and the incorporated radioactivity was determined by scintillation counting.

In time course experiments the MTase reactions contained ∼5 nM wild-type M.MpeI or ∼350 nM purified M.MpeI(Δ323A+N324G+R326G+E305N), 2 μM double-stranded oligonucleotide substrates and 5 μM [methyl-^3^H]-SAM. The incorporated radioactivity was determined by withdrawing 30 μl aliquots from a larger reaction mixture at 5, 10, 20 and 30 min. Measurements were performed in triplicate.

Reactions analyzing steady state kinetics of methyl transfer contained ∼5 nM wild-type M.MpeI or ∼350 nM M.MpeI(Δ323A+N324G+R326G+E305N), 5 μM [methyl-^3^H]-SAM and varying amounts of double-stranded oligonucleotide substrates. (Data obtained in triplicate were fitted to the Michaelis-Menten equation and analyzed using the Prism 3 and 10 programs (GraphPad).

### Bioinformatics tools

The PBD file containing atomic coordinates of the M.MpeI-DNA-AdoHcy complex (4dkj) was obtained from the RCSB PDB website (https://www.rcsb.org/). Three dimensional structures of mutant M.MpeI variants were generated using the I-TASSER platform (32). Protein structures were visualized by PyMOL (The PyMOL Molecular Graphics System, 230 Version 1.8 Schrödinger, LLC).

## Results

The M.MpeI MTase of *Malacoplasma penetrans* HF-2 (formerly *Mycoplasma penetrans* HF-2) was predicted to consist of 395 amino acids (protein_id="BAC44284.1) (14,15). In this work we used a C-terminally His-tagged variant of M.MpeI encoded by the plasmid pET28-MMpeI (15). Sequencing of pET28-MMpeI revealed that the N-terminal sequence of the wild-type MTase was Met-Gly-Asn-Ser (GenBank accession number: OR574779), rather than Met-Asn-Ser reported previously (https://www.ncbi.nlm.nih.gov/protein/4DKJ_A). Here, for consistency with previous publications, we ignored this one amino acid difference, and adhered to the original numbering of the residues (15).

### Sequence fidelity of wild-type M.MpeI

**Figure 1.**
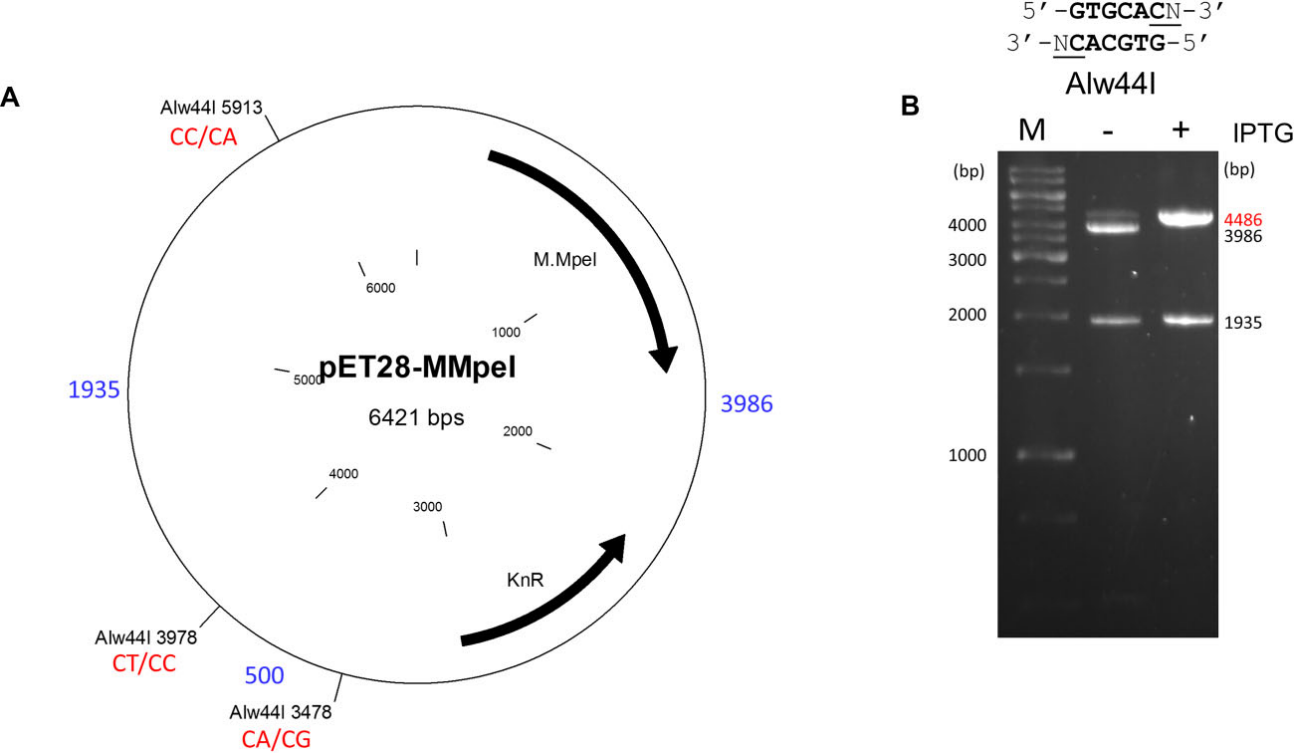
Testing the sequence specificity of wild-type M.MpeI by digesting the plasmid pET28-MMpeI with Alw44I. (**A**) Positions of the Alw44I cleavage sites, sizes of the fragments obtained after complete digestion (in bp, blue), and the CN sites (in red) created by the 3′-C of the Alw44I recognition sequence and the 3′-flanking nucleotide (see above the gel). (**B**) Fragment patterns of Alw44I digested plasmids purified from uninduced and IPTG induced cultures. Size of the protected fragment is shown in red. M, GeneRuler 1 kb DNA Ladder.

Unlike restriction enzymes, bacterial DNA MTases are thought not to be the subject of strong evolutionary pressure to maintain exquisite sequence specificity. Indeed, several MTases were shown to methylate, at lower rate, sequences that slightly differed from the canonical substrate site (33–36). Because the promiscuous activity can be exploited as starting point for creating altered specificity mutants (37,38), we first tested whether M.MpeI has detectable non-canonical activity. The plasmid pET28-MMpeI was purified from cells induced for M.MpeI expression and digested with three restriction enzymes (Alw44I, XmiI and BsuRI) known to be sensitive to C5-cytosine methylation in CA, CC or CT sequence context. Alw44I recognizes 5′-GTGCAC sites, but does not cut if the 3′-cytosine is methylated at least in one strand (5′-GTGCA^m5^C/5′-GTGCAC) (21) (Supplementary Table S4). The three Alw44I sites of pET28-MMpeI collectively represent all possible 3′ flanking nucleotides, thus digesting the plasmid with Alw44I can test methylation of all four possible CN dinucleotides (Figure 1). Upon induction of M.MpeI expression only one new band appeared in the Alw44I digest. The ∼4500 bp protected fragment indicated methylation at the Alw44I_3478_ site (Figure 1). This Alw44I site is flanked by 3′-A and 3′-G, thus the appearance of the 4486 bp protected fragment could indicate CA as well as CG specific methylation. However, the absence of the 5921 bp (1935 + 3986) and the 6421 bp (1935 + 3986 + 500) protected fragments suggested that the Alw44I_3478_ site was protected by CG rather than by CA specific methylation. CC specific methylation would produce 6421 and/or 5921 and/or 2435 bp fragments, whereas CT specific methylation would produce a 2435 bp fragment. None of these fragments were detected in the Alw44I digests (Figure 1). XmiI digestion is blocked when the 3′-cytosine of its recognition site is methylated on both strands (5′-GTMKA^m5^C/5′-GTMKA^m5^C) (21) (Supplementary Table S4). In pET28-MMpeI the XmiI site at position 1225 (Supplementary Figure S1) is flanked by 3′-A on both sides, thus absence of XmiI digestion of this site can detect CA specific methylation. BsuRI cleavage is blocked if the inner cytosine of the recognition site is methylated in at least one strand (5′-GG^m5^CC/5′-GGCC) (21) (Supplementary Table S4) allowing detection of ^m5^CC specific methylation by BsuRI digestion. There are 23 BsuRI sites in pET28-MMpeI. Neither the XmiI nor the BsuRI digestion of pET28-MMpeI produced protected fragments that would indicate non-canonical MTase activity of wild-type M.MpeI (Supplementary Figure S1). Considering that pET28-MMpeI was isolated from cells overproducing M.MpeI, these observations indicated that M.MpeI has very high sequence fidelity.

### Selected features of the M.MpeI-DNA recognition complex

**Figure 2.**
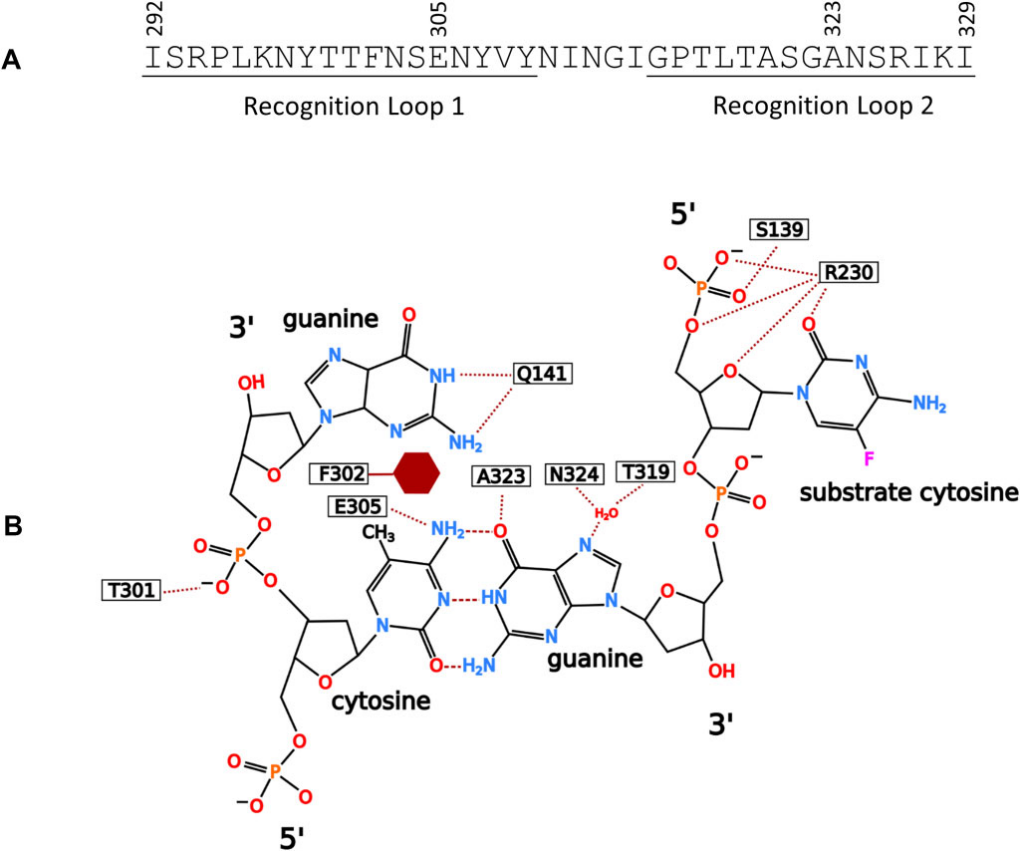
Selected features of sequence specific DNA recognition by M.MpeI as revealed by X-ray crystallography (15). (**A**) Amino acid sequence of the recognition loops. (**B**) Schematic representation of sequence specific interactions between the enzyme and the target site. Carbon 5 of the target cytosine carries a fluorine atom, whereas the cytosine in the non-substrate strand of the palindromic recognition site is methylated in the C5 position (15).

The X-ray structure of the specific complex formed by M.MpeI and a double-stranded oligonucleotide, in which the cytosine of the CG site in the non-substrate strand was methylated (5′-CG/5′-^m5^CG) revealed specific elements of the recognition mechanism (15). In the X-ray structure the place of the flipped-out target cytosine is occupied by the side chain of Gln141, which forms H-bonds with the orphan guanine (Figure 2). An interesting feature of the complex is the distortion of the helical structure caused by the aromatic side chain of Phe302, which intercalates between the ^m5^C and the G of the non-substrate strand (Figure 2). The characteristic deformation of the substrate site was suggested to act as an indirect readout element of the specific recognition mechanism (15).

In the crystal structure two short loops of the target recognizing domain appear to be highly important for recognition of the non-substrate base pair of the target sequence (5′-CG/5′-^m5^CG): Loop 1 and Loop 2 comprise residues Ile292 to Tyr309 and Gly315 to Ile329, respectively (Figure 2). Two amino acids, one from each loop, form specific H-bonds with the non-substrate C:G base pair of the recognition sequence. The carbonyl oxygen of the Glu305 side chain accepts an H-bond from N4 of the C5-methylcytosine in the non-substrate strand (5′-CG/5′-^m5^CG), whereas Ala323 donates, by its main-chain nitrogen, an H-bond to the guanine O6 in the substrate strand (5′-CG/5′-^m5^CG), (15) (Figure 2).

### Perturbing specific enzyme–DNA contacts by site directed mutagenesis

First, we tested whether replacing the intercalating Phe302 has any effect on M.MpeI sequence specificity. Five variants with substitutions of Phe302 were constructed: F302A/Q/M/W/Y. Alw44I digestion of the plasmids encoding the mutant MTases did not reveal non-canonical MTase activity, upon induction only the 4486 bp protected fragment resulting from CG specific methylation at Alw44I_3478_ appeared (Supplementary Figure S2).

CG specific activity of the variants was tested with Hin6I digestion. Hin6I has 46 sites in pET28-MMpeI, and does not cut if its recognition sequence is methylated on both strands (5′-G^m5^CGC/5′-G^m5^CGC (21)) (Supplementary Table S4). Variants with aromatic side chain (F302W/Y) showed high CG specific activity, whereas the activity of the Ala and Gln variants was barely detectable (Supplementary Figure S2).

Next we wished to perturb the base specific interactions between the MTase and the non-substrate base pair of the recognition sequence. Three replacements of Glu305 were constructed: E305A/S/Q. The mutant enzymes had CG specific activity (Hin6I digestion). Non-canonical activity was not detected (Alw44I digestion, Supplementary Figure S3). To further investigate the role of Recognition Loop 1, we created a deletion mutant, which lacked the segment extending from Thr301 to Glu305. In the X-ray structure Thr301 interacts with the sugar-phosphate backbone of the non-substrate strand (15) (Figure 2). The Δ[T301-E305] deletion mutant had very low CG specific activity. Non-canonical activity was not detectable (Supplementary Figure S4). In summary, the modifications created in Recognition Loop 1 alone did not result in altered MTase specificity.

**Figure 3.**
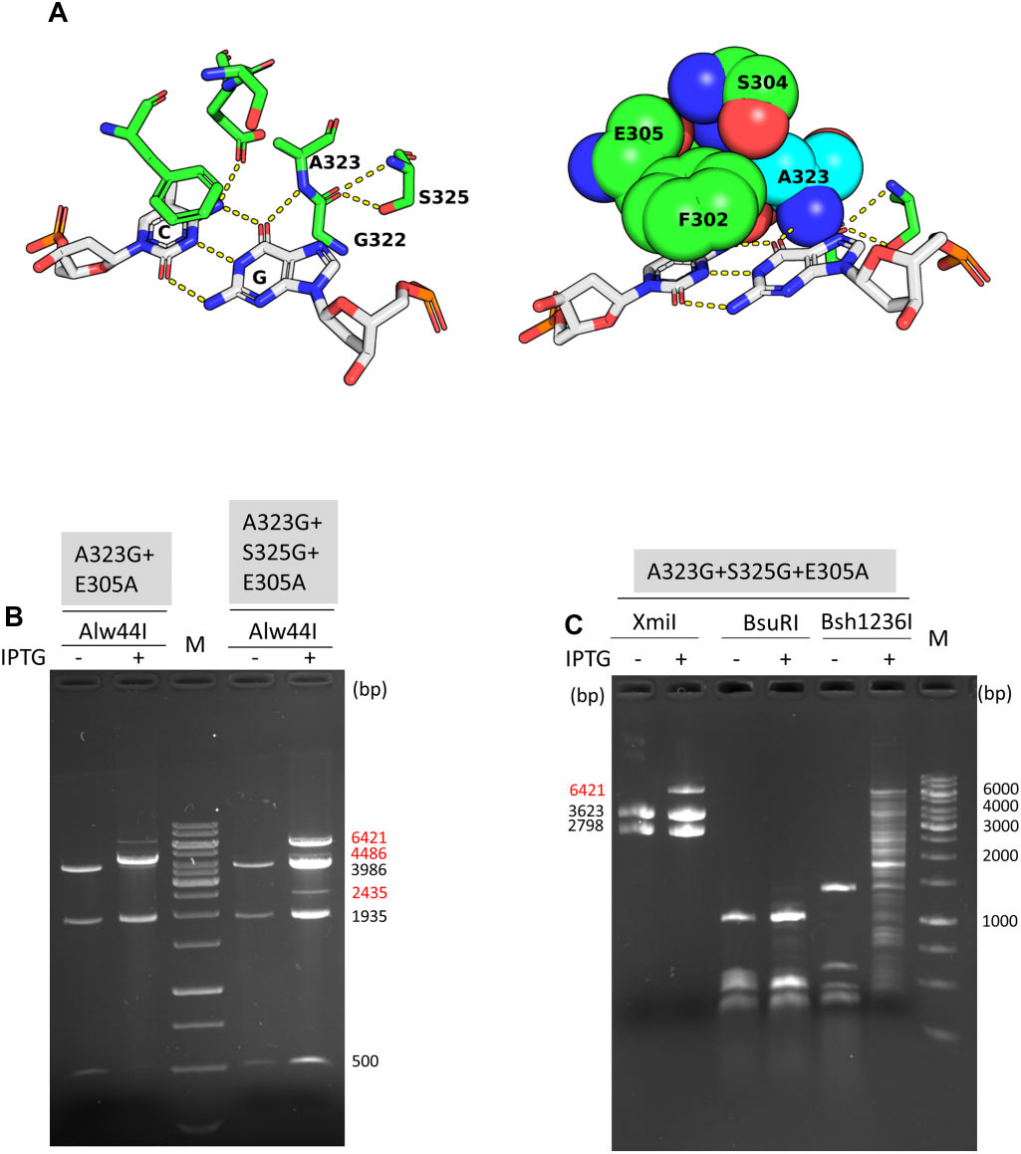
Methylation specificity of M.MpeI(A323G+E305A) and M.MpeI(A323G+S325G+E305A). (**A**) Interactions maintaining the stability of the H-bond between Ala323 and the guanine-O6 of the CG substrate site (15). Left panel, hydrogen bonds between the Ser325 and the oxygen of the peptide bond between Gly322 and Ala323; Right panel, pocket formed by Recognition Loop 1 residues holding the methyl group of Ala323. (**B**) Digestion of pET28-MMpeI(A323G+E305A) and pET28-MMpeI(A323G+S325G+E305A) with Alw44I. (**C**) Digestion of pET28-MMpeI(A323G+S325G+E305A) with XmiI, BsuRI and Bsh1236I. Fragment sizes in red letters indicate undigested fragments. For interpretation of the digestion patterns see the plasmid maps in Figure 1 and Supplementary Figure S1. M, GeneRuler 1 kb DNA Ladder.

The most important interaction between the 3′-half of the target site and Recognition Loop 2 appears to be the H-bond between the main-chain nitrogen of Ala323 and the guanine O6 in the substrate strand (5′-CG/5′-^5m^CG) (15) (Figure 2). Because the interaction is with the main chain, the options for rational substitutions of Ala323 are limited. Replacement of Ala323 with proline yielded an almost completely inactive variant, whereas the A323V variant had weak CG specific activity (not shown). In the crystal structure of the M.MpeI-DNA complex the NH group of the peptide bond between Gly322 and Ala323 is positioned optimally for the formation of a hydrogen bridge with the O6 atom of the guanine following the substrate cytosine. The favorable position appears to be maintained by two interactions (15) (Figure 3A):

- The methyl group of Ala323 fits into a small pocket formed by Phe302, Ser304 and Glu305 possibly limiting the mobility of Ala323.
- Ser325 forms hydrogen bridges with the oxygen of the peptide bond between Gly322 and Ala323.

We tried to destabilize the position of Ala323 by removing the Ala323 methyl group and/or the side chain of Ser325. To simultaneously perturb contacts to both bases of the non-substrate G:C base pair, the A323G and S325G substitutions were combined with the E305A mutation. Three mutants were constructed: A323G+E305A, S325G+E305A and A323G+S325G+E305A. Alw44I digestion revealed that the A323G+S325G+E305A triple mutant had significant non-CG specific activity (Figure 3B). The partial protection against XmiI digestion and the almost complete lack of protection against BsuRI digestion suggested that the non-CG specific activity of the A323G+S325G+E305A triple mutant was mainly CA specific (Figure 3C). The level of canonical CG specific methylation was assessed by Bsh1236I digestion. Bsh1236I cuts 5′-CGCG sites, but its activity is blocked by hemimethylation of either cytosine (5′-^m5^CGCG/5′-CGCG, 5′-CG^m5^CG/5′-CGCG) (21) (Supplementary Table S4). There are 35 Bsh1236I sites in pET28-MMpeI. The partial resistance to Bsh1236I indicated that the A323G+S325G+E305A triple mutant retained substantial CG specific MTase activity (Figure 3C).

In a previous study sequence specific contacts between the HhaI MTase and the 3′ base pair of its recognition sequence (5′-GCGC/5′-GCGC) were abolished by truncation of Recognition Loop 2 (39). The amino acid sequence similarity between the recognition loops of the two enzymes (Supplementary Figure S5) suggested that a similar approach might work for M.MpeI as well. We created microdeletions in the G322-N324 segment (Supplementary Figure S5). The variants with single amino acid deletions were either inactive (ΔG322), or had barely detectable activity (ΔA323 and ΔN324), whereas the variants carrying two or three amino acid deletions (Δ[A323-N324] and Δ[G322-N324]) had weak residual activity. Alw44I digestion of the plasmid expressing the Δ[A323-N324] variant produced faint bands indicating very weak non-CG specific methylation (Supplementary Figure S5).

**Figure 4.**
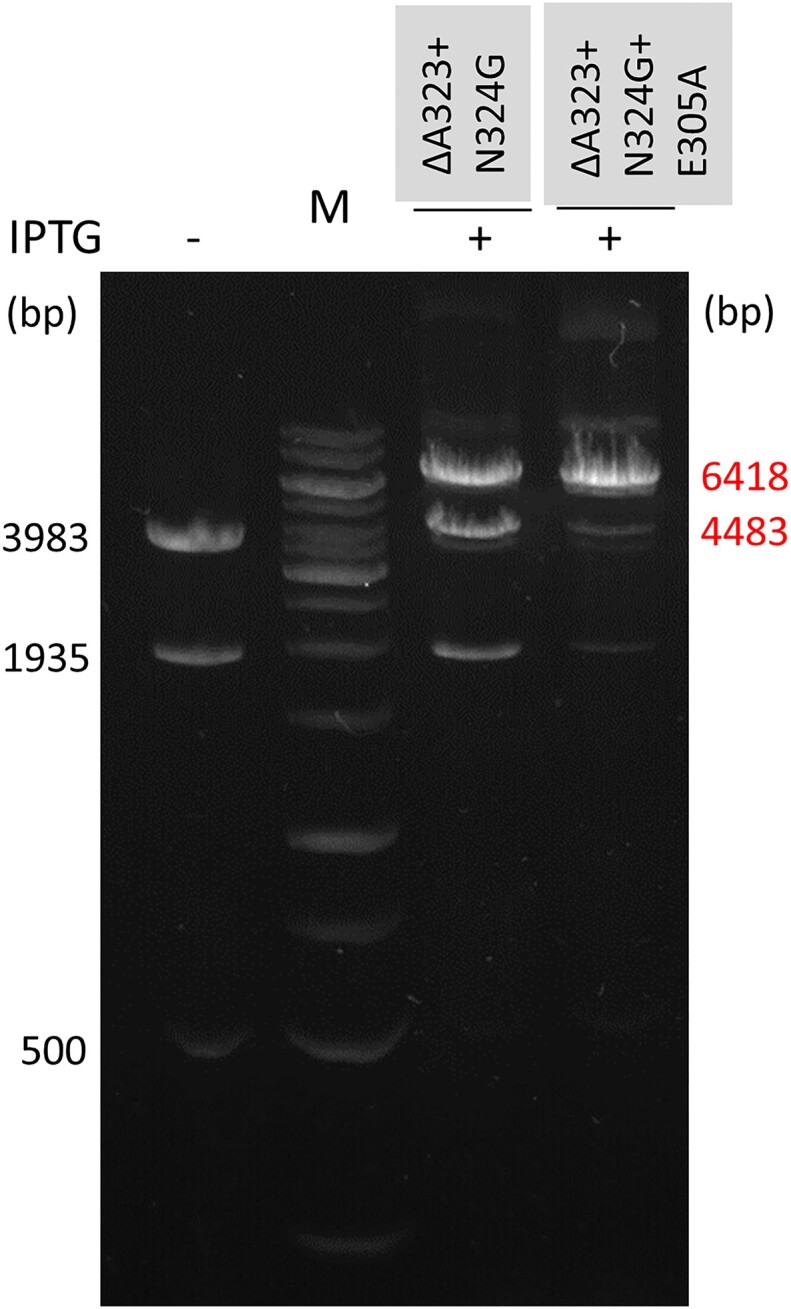
Alw44I digestion of pET28-MMpeI expressing the indicated M.MpeI variants. Sizes of the protected fragments are shown in red. For interpretation of the fragment pattern, see Figure 1. M, GeneRuler 1 kb DNA Ladder.

We hypothesized that the Δ[A323-N324] deletion mutant was more active than the ΔA323 single mutant because deletion of Ala323 caused the Asn324 side chain to adopt a position unfavorable for activity. To test this hypothesis, we generated a 3D model of the ΔA323 variant using the I-Tasser webserver (32). PyMOL alignment of the predicted M.MpeI(ΔA323) structure with the X-ray structure of wild-type M.MpeI showed that in the ΔA323 variant the Asn324 side chain indeed substantially deviated from its original position possibly displacing the side chain of Phe302 (Supplementary Figure S6). To abolish the assumed steric conflict, we replaced Asn324 with glycine. In the Alw44I digestion of the plasmid expressing the ΔA323+N324G double mutant the appearance of the strong band corresponding to the full-length linear plasmid (6418 bp) indicated substantial level of non-canonical methylation (Figure 4). To perturb recognition of both members of the non-substrate G:C base pair, the E305A mutation was introduced into the ΔA323+N324G variant. The ΔA323+N324G+E305A triple mutant showed even higher non-canonical activity than the double mutant: in the Alw44I digest the full-length plasmid became the dominant band (Figure 4).

**Figure 5.**
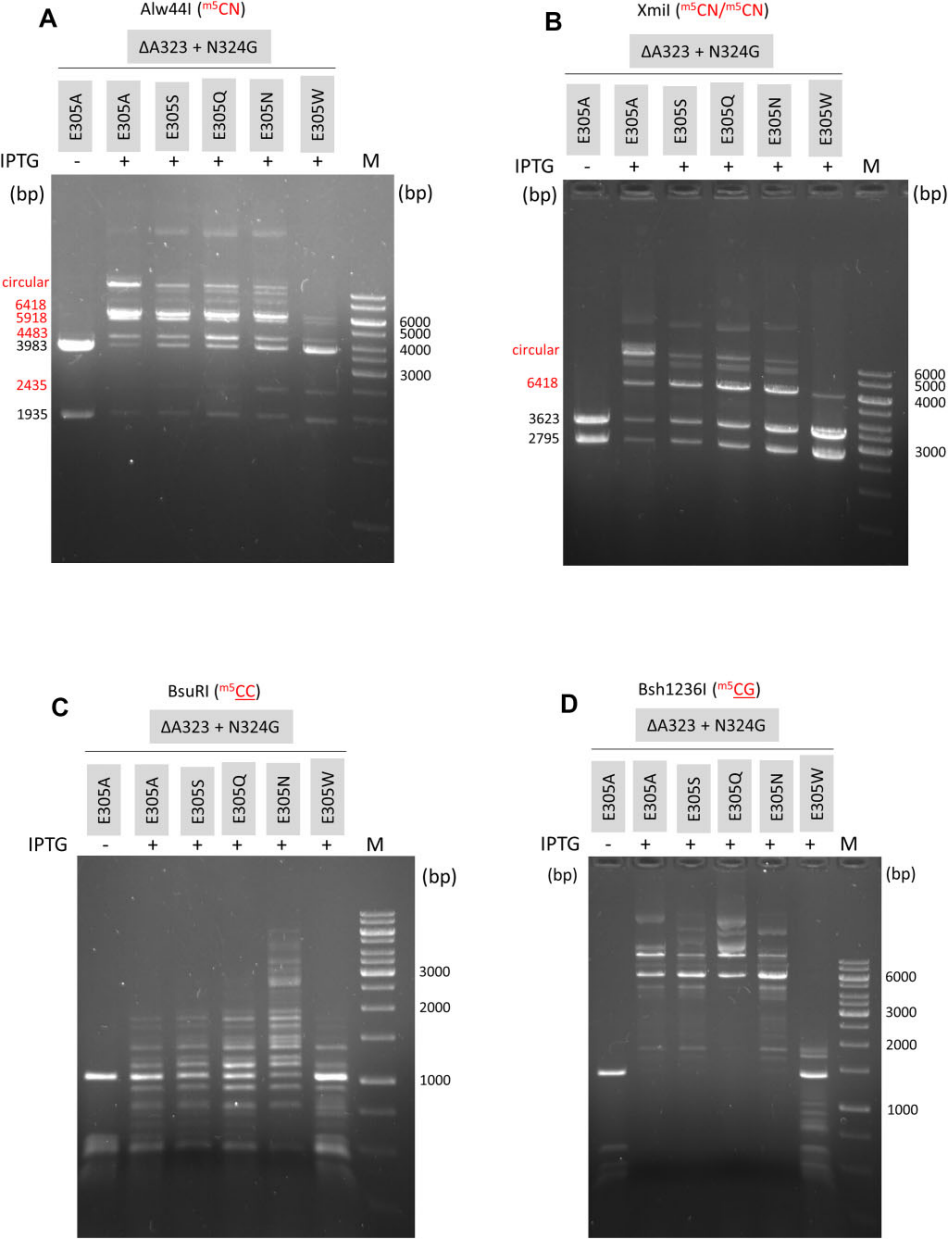
Digestion of pET28-MMpeI(ΔA323+N324G+E305X) plasmids with Alw44I, XmiI, BsuRI and Bsh1236I. On panels A and B sizes of the protected fragments are shown in red. For interpretation of the Alw44I and XmiI fragment pattern, see Figure 1 and Supplementary Figure S1, respectively. The plasmids contain 23 BsuRI and 35 Bsh1236I sites. The substrate site whose methylation status can be tested by digestion with the respective restriction enzyme is shown in red above the gels. M, GeneRuler 1 kb DNA Ladder.

To test whether the promiscuous activity of the ΔA323+N324G+E305A variant can be increased by inserting different amino acids at position 305, the codon specifying A305 was mutagenized using the randomized oligonucleotide AK809. Four mutants (ΔA323+N324G+E305S/Q/N/W) were characterized. The ΔA323+N324G+E305W variant had very low MTase activity (Figure 5), whereas the overall MTase activity of the ΔA323+N324G+E305S/Q/N variants was comparable with that of the parental ΔA323+N324G+E305A mutant. Alw44I and XmiI digestions suggested that the ΔA323+N324G+E305A/S/Q/N variants had significant CA specific activity (Figure 5A and B). BsuRI digestion revealed that they also had CC specific activity, which appeared to be the highest in the ΔA323+N324G+E305N enzyme (Figure 5C). The CG specific activity indicated by the resistance to Bsh1236I digestion appeared to be comparable between the E305A/S/Q/N derivatives (Figure 5D).

Three variants (ΔA323+N324G+E305A/N/W) were purified by affinity chromatography, and their substrate preference was assessed by *in vitro* MTase reaction using [methyl-^3^H]-labeled SAM and four double-stranded oligonucleotide substrates. The 23-mer duplexes had the same sequence except for the substrate CG/CA/CC/CT site in the middle (Supplementary Tables S2 and S6). Because the non-canonical CA/CC/CT sites are asymmetric and can be methylated only on one strand, for reliable comparison the CG site in the canonical substrate was synthesized to contain C5-methylated CG on one strand (AK702-703, Supplementary Tables S2 and S6). The results of the *in vitro* MTase reactions were consistent with the *in vivo* activities infered from plasmid digestions. The ΔA323+N324G+E305W variant had very low CG specific activity and no detectable activity with the other substrates. The ΔA323+N324G+E305A and ΔA323+N324G+E305N variants methylated CA as well as CC sites, but the CG specific activity was still dominant for both enzymes. No activity was detected with the CT substrate (Supplementary Table S5).

### Isolation of altered specificity mutants by random mutagenesis and directed evolution

We tried to further shift the specificity of the ΔA323+N324G+E305A triple mutant towards non-CG substrate sites by random mutagenesis and selection for plasmids, which acquired resistance to Alw44I digestion. First we mutagenized six positions of Recognition Loop 2 (amino acid positions 320 – 322 and 324 – 326) by inverse PCR using NNS-randomized oligonucleotides (AK793-794 and AK775-776, Supplementary Tables S2 and S3). The ratio of non-CG *vs*. CG specific MTase activity of the mutant enzymes obtained by Alw44I selection was not better than that of the parental triple mutant (not shown).

In the second approach a segment of the M.MpeI(ΔA323+N324G+E305A) gene extending from the beginning of the variable region to the 3′-end of the MTase coding sequence was mutagenized by error prone PCR. The mutant MTase gene library was cloned in the plasmid vector pOK-BAD (27), in which the gene of interest is transcribed from the arabinose inducible *E. coli araBAD* promoter. Sequencing of plasmids from six randomly selected clones revealed that mutations occured at a frequency of 2.5 mutations per gene with ∼80% being A to G transitions.

**Figure 6.**
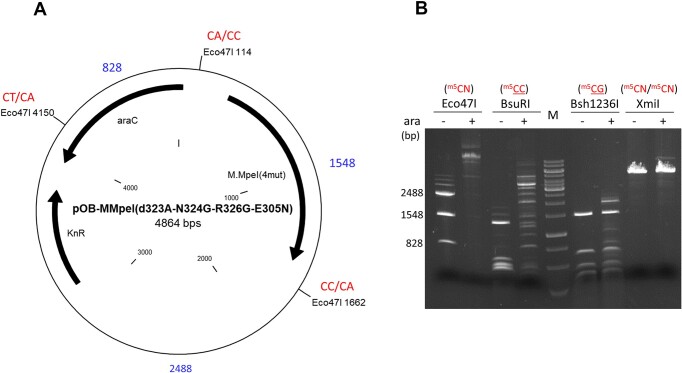
Testing the methylation specificity of M.MpeI(ΔA323+N324G+R326G+E305N). (**A**) Positions of Eco47I cleavage sites, the sizes of the fragments obtained after complete digestion (in bp, blue), and the CN sites (in red) created by the 3′-C of the Eco47I recognition sequence GGWCC and the 3′-flanking nucleotide. The unique XmiI site (not shown on the map) is flanked by adenine on both sides. (**B**) Digestion of pOB-MMpeI(ΔA323+N324G+R326G+E305N) with methylation sensitive restriction enzymes. Plasmid preparations marked by + signs were purified from cells induced with arabinose for five hours. The methylation specificity tested with the particular enzyme is shown in red above the enzyme's name. M, GeneRuler 1 kb DNA Ladder. The digestion patterns were reproduced in three independent experiments.

To select for plasmids expressing M.MpeI variants with non-CG MTase specificity, a sample of the plasmid preparation isolated from a mutagenized culture representing ∼60 000 clones was digested with Eco47I. Eco47I cuts 5′-GGWCC sites, but hemimethylation of the 3′-cytosine (5′-GGWC^m5^C/5′-GGWCC) blocks cleavage (21) (Supplementary Table S4). The pOK-BAD-based plasmids expressing the M.MpeI variants have three Eco47I sites. The three Eco47I sites and their 3′ flanking nucleotides represent all possible non-canonical substrate sites (CA, CC and CT), but lack the canonical CG site (Figure 6). Thus, Eco47I digestion can select for CH methylation specificity. Kn^R^ transformants obtained from the Eco47I-digested plasmid library were analyzed by digestions with Eco47I, BsuRI and Bsh1236I. We found one clone, whose non-CG specific activity was higher, and CG specific activity was lower than those of the parental M.MpeI(ΔA323+N324G+E305A) (Supplementary Figure S7). The MTase gene carried by this plasmid had two new point mutations: a silent mutation and a mutation resulting in R326G replacement in Recognition Loop 2.

Previously we observed that in the triple mutant M.MpeI(ΔA323+N324G+E305A) replacement of Ala305 with Asn resulted in increased CC specific MTase activity (Figure 5C). However, the CG specific activity of M.MpeI(ΔA323+N324G+E305N) was relatively high (Figure 5D). In the hope to combine the elevated CC activity and the low CG activity associated with the E305N and R326G mutations, respectively, we constructed the plasmid pOB-MMpeI(ΔA323+N324G+R326G+E305N). Digestion of the plasmid with BsuRI and Bsh1236I indicated relatively high CC specific and very low CG specific activity (Figure 6). The plasmid contains a single XmiI site flanked on both sides by adenine. The appearance of linear plasmid after XmiI digestion suggested that the mutant MTase had very low if any CA specific activity (Figure 6). The lack of protection of the XmiI site and the results of *in vitro* MTase measurements (see below) indicated that the M.MpeI(ΔA323+N324G+R326G+E305N) variant had very low activity on CA and no activity on CT sites. The almost complete protection against Eco47I digestion was at first glance surprising because only two of the three Eco47I sites overlap with CC sites, whereas the 3′ flanking nucleotides of the third Eco47I site are T and A (Figure 6). However, the Eco47I target sequence contains a CC dinucleotide (GGWCC), and C5-methylation of the inner cytosine (GGW^m5^CC) also blocks Eco47I cleavage (40,41), which explains why all three Eco47I sites were protected.

To test the *in vivo* activity of the evolved MTase variants in a different sequence context, the genes of the ΔA323+N324G+R326G+E305A and ΔA323+N324G+R326G+E305N variants were transferred from the pOB-based plasmids into pET28 vector. Digestions of pET28-MMpeI(ΔA323+N324G+R326G+E305A) and pET28-MMpeI(ΔA323+N324G+R326G+E305N) (Supplementary Figure S7) confirmed the conclusions drawn from digestions of the pOB-based plasmids:

- The dominant MTase activity was CC specific for both variants, and the A305N replacement increased the CC specific activity (BsuRI digestion)
- The CG specific activity was low, especially for the E305N mutant (Bsh1236I digestion)
- The CA and CT specific activities were very low or nonexistent (Alw44I and XmiI digestions)

Alw44I digestion of the pET28-based plasmids isolated from IPTG-induced cultures produced linear plasmid form, which indicates that one of the three Alw44I sites was cut (Supplementary Figure S7). This is consistent with the sequence contexts of the three Alw44I sites: two of the sites have one C as 3′ flanking nucleotide, whereas the third site is followed by A and G (Figure 1).

**Figure 7.**
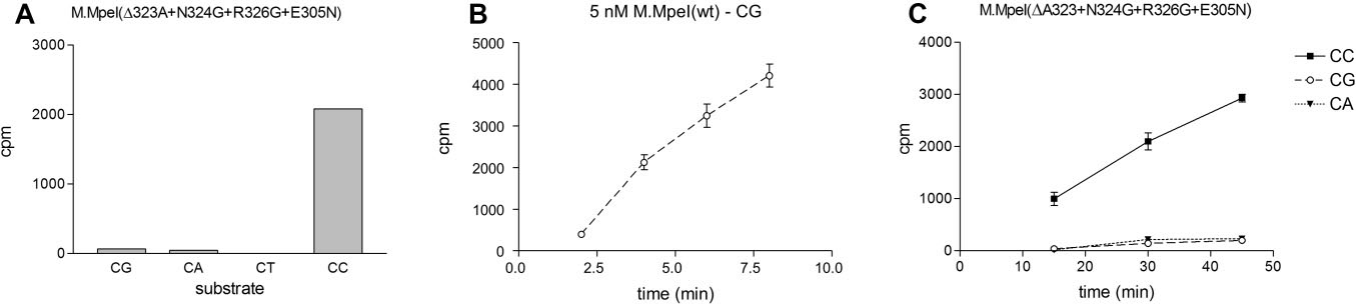
Testing the specificity of M.MpeI(ΔA323+N324G+R326G+E305N) by *in vitro* MTase assay using purified enzyme, [methyl-^3^H] labeled SAM and double stranded oligonucleotide substrates. In the substrate oligonucleotides (CG, AK702-AK703; CA, AK704-AK705; CT, AK706-AK707; CC, AK708-AK709) the substrate site was in AXXA context. (**A**) [methyl-^3^H] radioactivity incorporated by M.MpeI(ΔA323+N324G+R326G+E305N) in 30 min reactions. Average values of two independent experiments. (**B** and **C**) Time course of methyl transfer by wild-type M.MpeI and M.MpeI(ΔA323+N324G+R326G+E305N) into the CG, CA and CC duplexes. Average values of three independent experiments. The concentration of wild-type and mutant M.MpeI was 5 and 350 nM, respectively. Error bars: standard error of the mean.

M.MpeI(ΔA323+N324G+R326G+E305N) was purified from IPTG-induced *E. coli* ScarabXpress T7 *lac* cells by affinity chromatography (Supplementary Figure S8), and its substrate preference was tested *in vitro* using [methyl-^3^H]-labeled SAM and the double-stranded oligonucleotide substrates described above. Consistently with the *in vivo* data, the M.MpeI(ΔA323+N324G+R326G+E305N) variant predominantly methylated CC sites *in vitro*. It had very low activity on CG and CA sites and no activity on CT sites (Figure 7).

### Preference of M.MpeI(ΔA323+N324G+R326G+E305N) for flanking nucleotides

**Figure 8.**
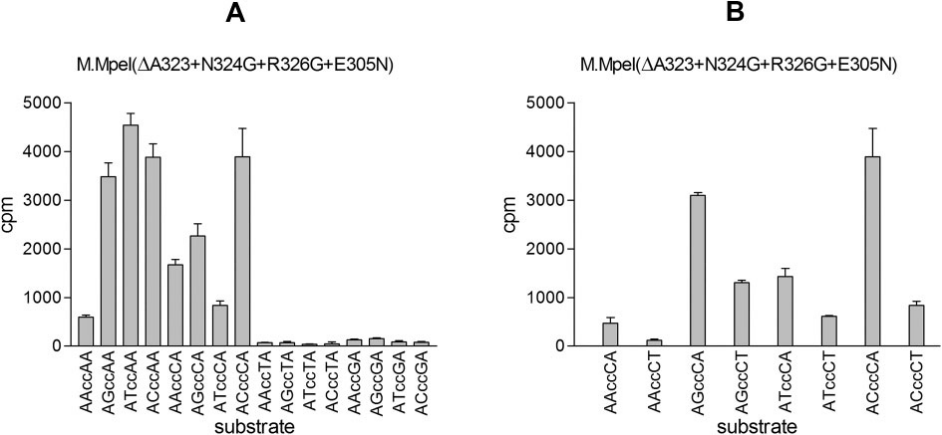
Effects of the flanking nucleotides on the CC-specific activity of M.MpeI(ΔA323+N324G+R326G+E305N). *In vitro* MTase reactions using purified enzyme, [methyl-^3^H] labeled SAM and double-stranded oligonucleotide substrates. [methyl-^3^H] radioactivity incorporated in 30 min reactions. Average values of three measurements. Two nucleotides preceding and following the CC substrate site are shown. To highlight the flanking nucleotides, the cc core substrate site is shown with lower case letters. Sequences of the substrate oligonucleotides are shown in Supplementary Tables S2 and S6. Error bars: standard error of the mean.

We tested whether M.MpeI(ΔA323+N324G+R326G+E305N) has any preference for the nucleotides bordering the CC substrate site. In *in vitro* MTase reactions using purified enzyme and double-stranded oligonucleotides that differed in the flanking nucleotides (Supplementary Table S6), CC sites followed by 3′ T or G were very poor substrates (Figure 8A). The preference for ccC was less clear than that for ccA because the ANccCA substrates also contained a CCA site (ANccCA) (To typographically distinguish the CC motif from the flanking nucleotides, in contexts focusing on flanking nucleotides the CC substrate site is shown in lower case). To address the preference for ccC sites, we tested the activity of the mutant enzyme with double-stranded oligonucleotides, in which the ccC site was followed by T rather than A (ANccCT, AK987 through AK994, Supplementary Table S6). The ANccCT substrates could also be methylated although less efficiently than their ANccCA counterparts (Figure 8B). These results confirmed the strong preference for A and a somewhat weaker preference for C in the 3′ flanking position.

In the 5′ flanking position all four nucleotides were accepted with A being the least preferred nucleotide (Figure 8B). These experiments revealed that the AK708-AK709 duplex (AAccAA), which we previously used as standard CC substrate, was methylated much less efficiently than duplexes containing AGccAA, ATccAA or ACccAA sites (Figure 8A and Supplementary Figure S9).

To test whether the strong bias regarding the 3′ flanking nucleotides can be detected *in vivo*, we purified pOB-MMpeI(ΔA323+N324G+R326G+E305N) plasmid DNA from uninduced and arabinose induced cells, and digested it with BsuRI, MspI, BamHI and NcoI. For all four restriction enzymes methylation of one strand of the recognition sequence is sufficient to block cleavage (21,42,43). The plasmid DNA isolated from induced cells was more protected against BsuRI than against MspI digestion. This is consistent with the *in vitro* data, because 12 of the 13 BsuRI sites in the plasmid have the preferred A or C as 3′ flanking nucleotide. In contrast, the CC motif of the MspI site is always followed by a 3′ G (Supplementary Figure S10A). The single BamHI site was digested, whereas the single NcoI site was almost completely protected in plasmids purified from arabinose induced cells. The difference is in line with the results of the *in vitro* MTase reactions described above because the CC motif of the BamHI site is followed by 3′ T and G, whereas the CC motif in the NcoI site is followed by 3′ A in both strands. One of the 5′-flanking nucleotides of the NcoI site is C, which allows efficient methylation (Supplementary Figure S10A). The preference for A or C in the 3′ flanking position was further confirmed by BamHI digestion of plasmids, which in addition to the original BamHI site, also contained a second engineered BamHI site flanked by A or C. The inserted BamHI site with 3′ flanking A was more protected against BamHI digestion than the site engineered to have 3′ flanking C (Supplementary Figure S10B), which suggests that ccA sites are better substrates than ccC sites.

In summary, *in vitro* as well as *in vivo* data show, that the quadruple mutant M.MpeI(ΔA323+N324G+R326G+E305N) predominantly methylates CC sites with a strong preference for A or C in the position following the CC motif (ccA or ccC). CC sites preceded by A (Acc) are methylated less efficiently than CC sites having G, C or T in the 5′ flanking position (G/C/Tcc).

**Figure 9.**
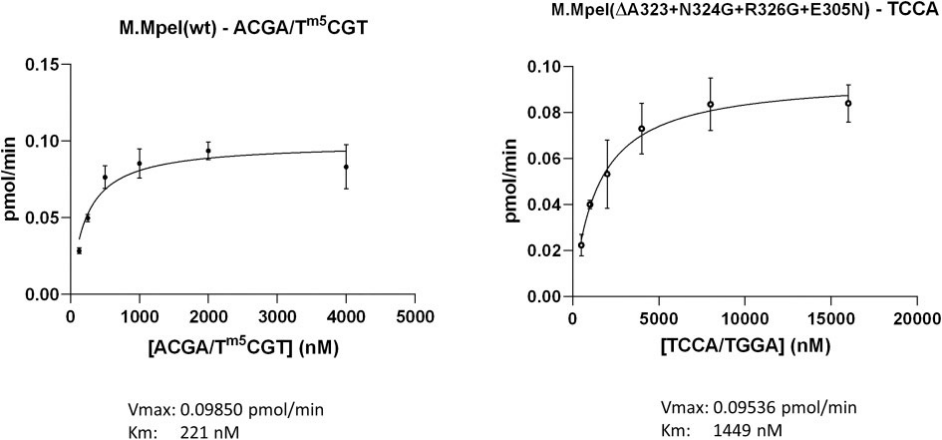
Steady-state kinetic analysis of methyl transfer into double stranded oligonucleotides containing the indicated substrate sites. The reactions contained 5 nM wild-type or 350 nM mutant M.MpeI and 2 μM 5′-ACGA/5′-T^m5^CGT (AK702–AK703) and 5′-TCCA/5′-TGGA (AK923-AK924) duplex, respectively. Error bars: standard error of the mean.

In steady state kinetics experiments wild-type M.MpeI methylated the hemimethylated 5′-CG/5′-^m5^CG substrate (AK702-AK703) with a K_M_ of ∼220 nM and a kcat of ∼0.65 min^−1^. The corresponding values for the quadruple mutant enzyme and the ATccAA substrate (AK923-AK924) were ∼1450 nM and ∼0.009 min^−1^, respectively (Figure 9). Thus, the kcat/Km value for the mutant enzyme and the ATccAA site was ∼470-fold lower, than the kcat/Km value for the wild-type enzyme and the canonical hemimethylated 5′-CG/5′-^m5^CG substrate.

## Discussion

At the beginning of this work we tested whether the CG specific M.MpeI can methylate non-CG sites. We could not detect any non-canonical activity. This result is consistent with previous bisulfite sequencing data on the sequence context of methylated cytosines in *E. coli* expressing M.MpeI (15). Given the short, 2 bp recognition sequence and the inherently low number of base specific contacts (15), the high degree of methylation specificity of M.MpeI is remarkable, and suggests that indirect readout mechanisms not readily recognizable in the X-ray model are essential for specific recognition of the target site.

Our original goal was to convert M.MpeI into an enzyme, which can methylate CA and/or CC and/or CT substrates, but cannot methylate CG sites. First we used site directed mutagenesis guided by the X-ray structure to eliminate specific contacts between the enzyme and the non-substrate base pair of the recognition sequence (5′-CG/5′-CG). Site directed mutagenesis was targeted to the two recognition loops, which appear to be primarily responsible for specific target recognition (15). Substitutions introduced into either one of the loops did not yield MTase with detectable non-CG specific activity. The first variant showing non-CG specificity was the triple mutant M.MpeI(A323G+S325G+E305A), which carried substitutions in both recognition loops presumably perturbing specific interactions with both members of the non-substrate base pair (5′-CG/5′-CG). The M.MpeI(A323G+S325G+E305A) variant had a mixture of CG and CA specific activity (Figure 3).

Deletion of Ala323 combined with the N324G substitution proved to be more effective in perturbing contacts to the guanine of the substrate strand (5′-CG/5′-CG) and relaxing canonical specificity than substitutions of Ala323: the ΔA323+N324G+E305A variant had higher relative level of non-CG activity than the A323G+S325G+E305A variant (Figure 4*vs*. Figure 3). The N324G replacement presumably eliminated an unfavorable distortion of the MTase structure created by the Ala323 deletion. Here again, the variant carrying replacements in both recognition loops (ΔA323+N324G+E305A), thus affecting both members of the non-substrate base pair (5′-CG/5′-CG) displayed higher non-CG activity than the one (ΔA323+N324G) carrying mutations only in Recognition Loop 2 (Figure 4).

The ratio of non-canonical *vs*. canonical activity was further increased by a single round directed enzyme evolution experiment involving random mutagenesis of the C-terminal ∼40% of M.MpeI encompassing the target recognition domain, and selection for variants methylating cytosines in CH dinucleotides (H stands for A or C or T). For the selection of altered specificity variants we used the method that is based on methylation of plasmids *in vivo* by the encoded MTase, and selection for plasmids that had become resistant to a restriction endonuclease blocked by ^m5^C methylation in the desired sequence context. This method, originally devised for cloning DNA MTase genes (44) and complete restriction-modification systems (45), was later adopted for the selection of mutant MTases (46,47) notably those with altered specificity (8,37–39,41,48). Great advantages of the method are the physical linkage between the mutant genes and their encoded phenotypes manifested in the methylation patterns of the plasmid, and the powerful selection provided by restriction digestion (3,49).

The evolved enzyme M.MpeI(ΔA323+N324G+R326G+E305A) carried a third mutation (R326G) in Recognition Loop 2, had the capacity to methylate non-CG sites, and its CG specific activity was substantially lower than that of the parental triple mutant (Supplementary Figure S7 versus Figure 5). A further site directed mutation (E305N) resulted in the quadruple mutant M.MpeI(ΔA323+N324G+R326G+E305N), which methylated CC sites, had very low activity on CG and CA sites and no detectable activity on CT sites (Figures 6 and 7). This final quadruple mutant showed very strong preference for A or C in the 3′ flanking position (5′-CCA or 5′-CCC, Figure 8), and moderate preference for G, C and T in the 5′ flanking position (Figure 8 and Supplementary Figure S9). Wild-type M.MpeI methylated CGA, CGG, CGC and CGT sites with comparable efficiencies (Supplementary Figure S12), thus recognition of the nucleotide following the CC „core’ target site appears to be result of the introduced mutations. The strong preference for 3′-flanking A or C makes M.MpeI(ΔA323+N324G+R326G+E305N) a MTase of CCA and CCC specificity.

The M.MpeI(ΔA323+N324G+R326G+E305N) mutant enzyme is a much less efficient MTase than wild-type M.MpeI (Figure 9). However, even this modest catalytic efficiency appears to be higher than that of the naturally evolved eukaryotic *de novo* MTase Dnmt3a (50).

Random mutagenesis coupled with selection for the evolved phenotype is considered a more efficient approach to obtain new specificity or function for proteins, than rational design (51). In the work decribed here the directed evolution step indeed greatly contributed to the shift in methylation specificity, but the preceding site-directed mutagenesis steps guided by the X-ray structure of the specific complex (15) proved very useful in eliminating the original enzyme-substrate interactions and ultimately leading to promiscuous MTase activity. Obviously, the observed changes in recognition specificity rest on multiple structural changes of the MTase, but the predicted structure of the quadruple mutant generated by the I-TASSER platform (32) suggests a possible mechanism for recognition of the guanine in the non-substrate base pair (5′-CC/5′-GG): the side-chain nitrogen of Asn305 is in good position to donate bifurcated H-bonds to the guanine (Supplementary Figure S11).

To our knowledge no CCA or CCC specific DNA MTase is known to exist (21), thus M.MpeI(ΔA323+N324G+R326G+E305N) represents novel MTase specificity.

## Supplementary Material

gkad1217_Supplemental_File

## Data Availability

The data underlying this article are available in GenBank at https://www.ncbi.nlm.nih.gov/genbank/ under accession code OR574779.
