# Supplementary material for: Conversion of the CG specific M.MpeI DNA methyltransferase into an enzyme predominantly methylating CCA and CCC sites
Source: Nucleic Acids Res. 2024 Jan 2;52(4):1896–908. doi: 10.1093/nar/gkad1217 (PMC10899764; doi:10.1093/nar/gkad1217)
Supplement: gkad1217_Supplemental_File [file gkad1217_supplemental_file.pdf]

Conversion of the CG specific MpeI DNA methyltransferase into an enzyme predominantly methylating CCA and CCC sites

Pál Albert, Bence Varga, Györgyi Ferenc and Antal Kiss

Supplementary material

RESULTS

Sequence fidelity of wild-type M.MpeI

The plasmid pET28-MMpeI was purified from cells induced for M.MpeI expression and digested with the restriction enzymes Alw44I, XmiI and BsuRI. For results of Alw44I digestion see main text. XmiI digestion is blocked if the 3'-cytosines of the recognition site are methylated on both strands (5'-GTMKA^{m5}C/5'-GTMKA^{m5}C) (1). There are two XmiI sites in pET28-MMpeI. The site at position 1225 is flanked on both sides by 3'-A, thus XmiI digestion of this site can detect CA specific methylation. No protection was detected at either of the XmiI sites (Supplementary Figure S1).

BsuRI cleavage is blocked if the inner cytosine of the recognition site is methylated at least on one strand (5'-GG^{m5}CC/5'-GGCC) (1), thus BsuRI digestion can detect ^{m5}CC specific methylation. There are 23 BsuRI sites in the plasmid. No difference was observed between the BsuRI patterns before and after induction (Supplementary Figure S1) suggesting that no ^{m5}CC specific methylation occured.

Supplementary Table S1. Plasmids used in this work.

**Plasmid name**[1]
pOK-BAD[2]
pET28-MMpeI[2]
pET28-MMpeI(F302A)
pET28-MMpeI(F302M)
pET28-MMpeI(F302Q)
pET28-MMpeI(F302Y)
pET28-MMpeI(F302W)
pET28-MMpeI(E305A)
pET28-MMpeI(E305S)
pET28-MMpeI(E305Q)

pET28-MMpeI(A323P)
pET28-MMpeI(A323V)
pET28-MMpeI(Δ[T301-E305])
pET28-MMpeI(ΔG322)
pET28-MMpeI(ΔA323)
pET28-MMpeI(ΔN324)
pET28-MMpeI(Δ[G322-A323])
pET28-MMpeI(Δ[G322-N324])
pET28-MMpeI(ΔA323+N324G)
pET28-MMpeI(ΔA323+N324G+E305A)
pET28-MMpeI(A323G+E305A)
pET28-MMpeI(S325G+E305A)
pET28-MMpeI(ΔA323+N324G+E305S)
pET28-MMpeI(ΔA323+N324G+E305Q)
pET28-MMpeI(ΔA323+N324G+E305N)
pET28-MMpeI(ΔA323+N324G+E305W)
pOB-MMpeI
pOB-MMpeI(ΔA323)
pOB-MMpeI(ΔA323+N324G+E305A)
pOB-MMpeI(ΔA323+N324G+R326G+E305A)
pOB-MMpeI(ΔA323+N324G+R326G+E305N)
pOB-MMpeI(ΔA323+N324G+R326G+E305N)-AK946
pOB-MMpeI(ΔA323+N324G+R326G+E305N)-AK948

[1]The plasmids are named to indicate the vector (pET28 or pOK-BAD) and the encoded M.MpeI variant. [2]The plasmids pOK-BAD and pET28-MMpeI were described in (2) and (3), respectively. All other plasmids were created in this work. All plasmids listed in the table confer kanamycin resistance.

Supplementary Table S2. Oligonucleotides used in this work

Name	Sequence 5' – 3'	Features, use
AK463	CCATACCATGGGCAATAG	Forward primer for PCR amplification of the M.MpeI gene. NcoI site (underlined)
AK514	GCGAACAGTGAAAACTATGTATACAAT	Forward primer for creating the F302A mutation (underlined)
AK515	AGTTGTGTAATTTTTAAGAGGTCTT	Reverse primer for the construction of F302A, F302M, F302Q, F302Y and F302W mutants
AK541	AACTATGTATACAATATCAATG	Forward primer for the construction of E305A, E305S, ΔA323+N324G+E305Q, ΔA323+N324G+E305N, ΔA323+N324G+R326G+E305N, and the Δ[T301-E305] mutants
AK542[1]	TGTGTAATTTTTAAGAGGTC	Reverse primer for the construction of the Δ[T301-E305] mutant
AK564	CGCACTGTTAAAAGTTGTG	Reverse primer for creating the E305A mutation (underlined)
AK565	TGGAACAGTGAAAACTATGTATACAAT	Forward primer for creating the F302W mutation (underlined)
AK566	ATGAACAGTGAAAACTATGTATACAAT	Forward primer for creating the F302M mutation, (underlined)
AK567	CAGAACAGTGAAAACTATGTATACAAT	Forward primer for creating the F302Q mutation, (underlined)
AK568	TATAACAGTGAAAACTATGTATACAAT	Forward primer for creating the F302Y mutation, (underlined)
AK579	CTGACTGTTAAAAGTTGTG	Reverse primer for creating the E305Q mutation (underlined)
AK580	CCGAACTCAAGAATTAAAATTGAAAC	Forward primer for creating the A323P mutation (underlined)
AK581	ACCAGATGCAGTAAGAGTTG	Reverse primer for the construction of the A323P, ΔA323+N324G, A323G+S325G+E305A and the S325G+E305A mutants
AK586	GCTACTGTTAAAAGTTGTG	Reverse primer for creating the E305S mutation (underlined)
AK603	GGTTCAAGAATTAAAATTGAAACTCAAC	Forward primer for creating the N324G mutation (underlined)
AK702	GAATTATAAAA**CG**AAAATAAAAC	Complementary oligonucleotides used in the *in vitro* MTase assay for testing CG specific activity. The substrate site is shown in bold blue.
AK703	GTTTTATTTT^{m5}CGTTTTATAATTC	
AK704	GAATTATAAAA**CA**AAAATAAAAC	Complementary oligonucleotides used in the *in vitro* MTase assay. The substrate site is shown in bold blue.
AK705	GTTTTATTTTTGTTTTATAATTC	
AK706	GAATTATAAAA**CT**AAAATAAAAC	Complementary oligonucleotides used in the *in vitro* MTase assay. The substrate site is shown in bold blue.
AK707	GTTTTATTTTAGTTTTATAATTC	
AK708	GAATTATAAAA**CC**AAAATAAAAC	Complementary oligonucleotides used in the *in vitro* MTase assay. The substrate site is shown in bold blue.
AK709	GTTTTATTTTGGTTTTATAATTC	
AK714	GAGATGATTATCTAGAAAAAACAGG	Forward primer for error-prone PCR, contains a XbaI site (underlined)

AK715	GGTGCTCGAGTTCCTC	Reverse primer for error-prone PCR, contains a XhoI site (underlined)
AK739	CGTAAGCTTGGCTTTGTTAGCAGC	Reverse primer for PCR amplification of the M.MpeI gene. HindIII site (underlined).
AK775	CTGCATCTGGT**NNSNNSNNS**ATTAAAATTGAAACTCAACAAGGTG	Forward primer for random mutagenesis of residues N324-R326 in Recognition Loop 2 of the ΔA323+N324G+E305A variant. The three randomized codons are shown in bold.
AK776	TAAGAGTTGGCCCAATACCATTGATATTG	Reverse primer used together with AK775.
AK793	GCCAACTCTTACT**NNSNNSNNS**GGTTCAAGAATTAAAATTGAAACTCAAC	Forward primer for random mutagenesis of residues A320-G322 in Recognition Loop 2 of the ΔA323+N324G+E305A variant. The three randomized codons are shown in bold.
AK794	CCAATACCATTGATATTGTATACATAGTTCGC	Reverse primer used together with AK793.
AK800	GGCAACGGCAGAATTAAAATTGAAACTCAACAAG	Forward primer for the construction of the A323G+S325G+E305A mutant (G323 and G325 codons underlined)
AK807	GCAAACGGCAGAATTAAAATTGAAACTCAAC	Forward primer for the construction of S325G+E305A mutant (G325 codon underlined)
AK808	GTTACTGTTAAAAGTTGTG	Reverse primer for the construction of ΔA323+N324G+E305N and ΔA323+N324G+R326G+E305N (reverse complement to N305 underlined)
AK809	CAACTTTTAACAGT**NNS**AACTATGTATACAATATCAATGG	Forward primers for site saturation mutagenesis of amino acid position 305. The randomized codon is shown in bold.
AK810[1]	TGTAATTTTTAAGAGGTCTTGAAATGATG	Reverse primer used with AK809.
AK921	GAATTATAAAG**CC**AAAATAAAAC	Complementary sequences. Substrate for testing the effect of flanking nucleotides on CC specific activity *in vitro*. The substrate site is shown in bold blue.
AK922	GTTTTATTTTGGCTTTATAATTC	
AK923	GAATTATAAAT**CC**AAAATAAAAC	Complementary sequences. Substrate for testing the effect of flanking nucleotides on CC specific activity *in vitro*. The substrate site is shown in bold blue.
AK924	GTTTTATTTTGGATTTATAATTC	
AK925	GAATTATAAAC**CC**AAAATAAAAC	Complementary sequences. Substrate for testing the effect of flanking nucleotides on CC specific activity *in vitro*. The substrate site is shown in bold blue.
AK926	GTTTTATTTTGGGTTTATAATTC	
AK927	GAATTATAAAA**CC**TAAATAAAAC	Complementary sequences. Substrate for testing the effect of flanking nucleotides on CC specific activity *in vitro*. The substrate site is shown in bold blue.
AK928	GTTTTATTTAGGTTTTATAATTC	
AK929	GAATTATAAAA**CC**GAAATAAAAC	Complementary sequences. Substrate for testing the effect of flanking nucleotides on CC specific activity *in vitro*. The substrate site is shown in bold blue.
AK930	GTTTTATTTCGGTTTTATAATTC	
AK931	GAATTATAAAA**CC**CAAATAAAAC	Complementary sequences. Substrate for testing the effect of flanking nucleotides on CC specific activity *in vitro*. The substrate site is shown in bold blue.
AK932	GTTTTATTTGGGTTTTATAATTC	
AK946	CCATCTGCGTTGGATCCAACCG	Complementary sequences with Eco31I- and Psp1406I-compatible overhangs. BamHI site underlined.
AK947	CGCGGTTGGATCCAACGCAG	
AK948	CCATCTGCGTGGGATCCCACCG	Complementary sequences with Eco31I- and Psp1406I-compatible overhangs. BamHI site underlined.
AK949	CGCGGTGGGATCCCACGCAG	

AK967	GAATTATAAAG**CC**TAAATAAAAC	Complementary sequences. Substrate for testing the effect of flanking nucleotides on CC specific activity *in vitro*. The substrate site is shown in bold blue.
AK968	GTTTTATTTAGGCTTTATAATTC	
AK969	GAATTATAAAG**CC**CAAATAAAAC	Complementary sequences. Substrate for testing the effect of flanking nucleotides on CC specific activity *in vitro*. The substrate site is shown in bold blue.
AK970	GTTTTATTTGGGCTTTATAATTC	
AK971	GAATTATAAAG**CC**GAAATAAAAC	Complementary sequences. Substrate for testing the effect of flanking nucleotides on CC specific activity *in vitro*. The substrate site is shown in bold blue.
AK972	GTTTTATTTCGGCTTTATAATTC	
AK973	GAATTATAAAT**CC**GAAATAAAAC	Complementary sequences. Substrate for testing the effect of flanking nucleotides on CC specific activity *in vitro*. The substrate site is shown in bold blue.
AK974	GTTTTATTTCGGATTTATAATTC	
AK975	GAATTATAAAT**CC**CAAATAAAAC	Complementary sequences. Substrate for testing the effect of flanking nucleotides on CC specific activity *in vitro*. The substrate site is shown in bold blue.
AK976	GTTTTATTTGGGATTTATAATTC	
AK977	GAATTATAAAT**CC**TAAATAAAAC	Complementary sequences. Substrate for testing the effect of flanking nucleotides on CC specific activity *in vitro*. The substrate site is shown in bold blue.
AK978	GTTTTATTTAGGATTTATAATTC	
AK979	GAATTATAAAC**CC**GAAATAAAAC	Complementary sequences. Substrate for testing the effect of flanking nucleotides on CC specific activity *in vitro*. The substrate site is shown in bold blue.
AK980	GTTTTATTTCGGGTTTATAATTC	
AK981	GAATTATAAAC**CC**TAAATAAAAC	Complementary sequences. Substrate for testing the effect of flanking nucleotides on CC specific activity *in vitro*. The substrate site is shown in bold blue.
AK982	GTTTTATTTAGGGTTTATAATTC	
AK983	GAATTATAAAC**CC**CAAATAAAAC	Complementary sequences. Substrate for testing the effect of flanking nucleotides on CC specific activity *in vitro*. The substrate site is shown in bold blue.
AK984	GTTTTATTTGGGGTTTATAATTC	
AK987	GAATTATAAAA**CC**CTAATAAAAC	Complementary sequences. Substrate for testing the effect of flanking nucleotides on CC specific activity *in vitro*. The substrate site is shown in bold blue.
AK988	GTTTTATTAGGGTTTTATAATTC	
AK989	GAATTATAAAG**CC**CTAATAAAAC	Complementary sequences. Substrate for testing the effect of flanking nucleotides on CC specific activity *in vitro*. The substrate site is shown in bold blue.
AK990	GTTTTATTAGGGCTTTATAATTC	
AK991	GAATTATAAAT**CC**CTAATAAAAC	Complementary sequences. Substrate for testing the effect of flanking nucleotides on CC specific activity *in vitro*. The substrate site is shown in bold blue.
AK992	GTTTTATTAGGGATTTATAATTC	
AK993	GAATTATAAAC**CC**CTAATAAAAC	Complementary sequences. Substrate for testing the effect of flanking nucleotides on CC specific activity *in vitro*. The substrate site is shown in bold blue.
AK994	GTTTTATTAGGGGTTTATAATTC	
AK1008	GAATTATAAAA**CG**CAAATAAAAC	Complementary sequences. Substrate for testing the 3'-flanking nucleotide preference of wild-type M.MpeI *in vitro*. The substrate site is shown in bold blue.
AK1009	GTTTTATTTG^{m5}CGTTTTATAATTC	
AK1010	GAATTATAAAA**CG**GAAATAAAAC	Complementary sequences. Substrate for testing the 3'-flanking nucleotide preference of wild-type M.MpeI *in vitro*. The substrate site is shown in bold blue.
AK1011	GTTTTATTTC^{m5}CGTTTTATAATTC	
AK1012	GAATTATAAAA**CG**TAAATAAAAC	Complementary sequences. Substrate for testing the 3'-flanking nucleotide preference of wild-type M.MpeI *in vitro*. The substrate site is shown in bold blue.
AK1013	GTTTTATTTA^{m5}CGTTTTATAATTC	

[1]The oligonucleotides AK542 and AK810 were designed using the published sequence of the M.MpeI gene (https://www.ncbi.nlm.nih.gov/protein/4DKJ_A). We noticed later that in pET28-

MMpeI the codon specifying Pro296 is CCC rather than CCT. Thus the underlined A should be G. The mismatch notwithstanding the oligonucleotides worked in the mutagenesis as expected.

Supplementary Table S3. Oligonucleotides used to create site directed mutations by inverse PCR.

**Mutation**	**Oligonucleotides**
F302A	AK514, AK515
F302M	AK566, AK515
F302Q	AK567, AK515
F302Y	AK568, AK515
F302W	AK565, AK515
E305A	AK564, AK541
E305S	AK586, AK541
E305Q	AK579, AK541
Δ[T301-E305]	AK541, AK542
A323P	AK580, AK581
N324G	AK603, AK581
A323G+S325G+E305A	AK800, AK581
S325G+E305A	AK807, AK581
ΔA323+N324G+E305N and ΔA323+N324G+R326G+E305N	AK541, AK808
position 305, random	AK809, AK810
positions 320-322, randomized	AK793, AK794
positions 324-326, randomized	AK775, AK776

For sequence of the oligonucleotides see Supplementary Table S2.

Supplementary Table S4. Restriction enzymes used to test methylation specificity of the M.MpeI variants *in vivo.*

Restriction enzyme	Recognition sequence	^{m5}C-methylation blocking cleavage[1]	Testable methylation specificity
Alw44I	GTGCAC<br>CACGTG	GTGCA<u>C</u><br>CACGTG	<u>C</u>N
BamHI	GGATCC<br>CCTAGG	GGAT<u>C</u>C<br>CCTAGG	<u>C</u>C
Bsh1236I	CGCG<br>GCGC	<u>C</u>GCG<br>GCGC<br><br>CG<u>C</u>G<br>GCGC	<u>C</u>G
BsuRI	GGCC<br>CCGG	GG<u>C</u>C<br>CCGG	<u>C</u>C
Eco47I	GGWCC<br>CCWGG	GGWC<u>C</u><br>CCWGG	<u>C</u>N
Hin6I	GCGC<br>CGCG	G<u>C</u>GC<br>CG<u>C</u>G	<u>C</u>G
MspI	CCGG<br>GGCC	<u>C</u>CGG<br>GGCC	<u>C</u>C
NcoI	CCATGG<br>GGTACC	<u>C</u>CATGG<br>GGTACC	<u>C</u>C
XmiI	GTMKAC<br>CAKMTG	GTMKA<u>C</u><br><u>C</u>AKMTG	<u>C</u>N

[1]Only the minimal requirement for restriction protection (in most cases, methylation of one strand) is shown. Data for methylation sensitivity of restriction enzymes were taken from REBASE (1). C5-methylated cytosines are underlined. M: A or C; K: G or T; W: A or T.

Supplementary Table S5. Substrate preference of M.MpeI(ΔA323+N324G+E305A/N/W) variants.

substrate[1]	+305A	+305N	+305W
AK702-AK703 (5’-CG/5’-^{m5}CG)	4961	940	307
AK704-AK705 (5’-CA/5’-TG)	3289	417	0
AK706-AK707 (5’-CT/5’-AG)	0	0	0
AK708-AK709 (5’-CC/5’-GG)	2351	429	0

[1]Sequence of the oligonucleotide duplexes:

AK702-703
5'-GAATTATAAAACGAAAATAAAAC-3'
3'-CTTAATATTTTGCTTTTATTTTG-5'
CH_3

AK704-705
5'-GAATTATAAAACAAAAATAAAAC-3'
3'-CTTAATATTTTGTTTTTATTTTG-5'

AK706-707
5'-GAATTATAAAACTAAAATAAAAC-3'
3'-CTTAATATTTTGATTTTATTTTG-5'

AK708-709
5'-GAATTATAAAACCAAAATAAAAC-3'
3'-CTTAATATTTTGGTTTTATTTTG-5'

The table shows [methyl-^{3}H] radioactivity (cpm) incorporated into double-stranded oligonucleotide substrates by purified M.MpeI(ΔA323+N324G+E305A/N/W) variants. MTase activity was measured in 30 min reactions as described in Materials and Methods. Average values of two independent experiments.

Supplementary Table S6. Double-stranded oligonucleotide substrates used to test the effects of flanking nucleotides on the CC specific activity of M.MpeI(ΔA323+N324G+R326G+E305N), and on the CG specific activity of wild-type M.MpeI**.**

Substrate site with four flanking nucleotides	Names of oligonucleotides	Sequence of the oligonucleotide duplex[1]
AAcgAA	AK702 – AK703	GAATTATAAAA**CG**AAAATAAAAC<br>CTTAATATTTTGCTTTTATTTTG
AAccAA	AK708 – AK709	GAATTATAAAA**CC**AAAATAAAAC<br>CTTAATATTTTGGTTTTATTTTG
AGccAA	AK921 - AK922	GAATTATAAAG**CC**AAAATAAAAC<br>CTTAATATTTCGGTTTTATTTTG
ATccAA	AK923 - AK924	GAATTATAAAT**CC**AAAATAAAAC<br>CTTAATATTTAGGTTTTATTTTG
ACccAA	AK925 - AK926	GAATTATAAAC**CC**AAAATAAAAC<br>CTTAATATTTGGGTTTTATTTTG
AAccCA	AK931 - AK932	GAATTATAAAA**CC**CAAATAAAAC<br>CTTAATATTTTGGGTTTATTTTG
AGccCA	AK969 - AK970	GAATTATAAAG**CC**CAAATAAAAC<br>CTTAATATTTCGGGTTTATTTTG

ATccCA	AK975 - AK976	GAATTATAAAT**CC**CAAATAAAAC<br>CTTAATATTTAGGGTTTATTTTG
ACccCA	AK983 - AK984	GAATTATAAAC**CC**CAAATAAAAC<br>CTTAATATTTGGGGTTTATTTTG
AAccTA	AK927 - AK928	GAATTATAAAA**CC**TAAATAAAAC<br>CTTAATATTTTGGATTTATTTTG
AGccTA	AK967 - AK968	GAATTATAAAG**CC**TAAATAAAAC<br>CTTAATATTTCGGATTTATTTTG
ATccTA	AK977 - AK978	GAATTATAAAT**CC**TAAATAAAAC<br>CTTAATATTTAGGATTTATTTTG
ACccTA	AK981 - AK982	GAATTATAAAC**CC**TAAATAAAAC<br>CTTAATATTTGGGATTTATTTTG
AAccGA	AK929 - AK930	GAATTATAAAA**CC**GAAATAAAAC<br>CTTAATATTTTGGCTTTATTTTG
AGccGA	AK971 - AK972	GAATTATAAAG**CC**GAAATAAAAC<br>CTTAATATTTCGGCTTTATTTTG
ATccGA	AK973 - AK974	GAATTATAAAT**CC**GAAATAAAAC<br>CTTAATATTTAGGCTTTATTTTG
ACccGA	AK979 - AK980	GAATTATAAAC**CC**GAAATAAAAC<br>CTTAATATTTGGGCTTTATTTTG
AAccCT	AK987 – AK988	GAATTATAAAA**CC**CTAATAAAAC<br>CTTAATATTTTGGGATTATTTTG
AGccCT	AK989 – AK990	GAATTATAAAG**CC**CTAATAAAAC<br>CTTAATATTTCGGGATTATTTTG
ATccCT	AK991 –AK992	GAATTATAAAT**CC**CTAATAAAAC<br>CTTAATATTTAGGGATTATTTTG
ACccCT	AK993 – AK994	GAATTATAAAC**CC**CTAATAAAAC<br>CTTAATATTTGGGGATTATTTTG
AAcgCA	AK1008 – AK1009	GAATTATAAAA**CG**CAAATAAAAC<br>CTTAATATTTTG<u>C</u>GTTTATTTTG
AAcgGA	AK1010 - AK1011	GAATTATAAAA**CG**GAAATAAAAC<br>CTTAATATTTTG<u>C</u>CTTTATTTTG
AAcgTA	AK1012 - AK1013	GAATTATAAAA**CG**TAAATAAAAC<br>CTTAATATTTTG<u>C</u>ATTTATTTTG

[1]The substrate site is shown in bold blue. In the duplexes AK702-AK703, AK1008-AK1009, AK1010-AK1011 and AK1012-AK1013 the bottom strand of the CG substrate site contains C5-methylated cytosine (underlined).

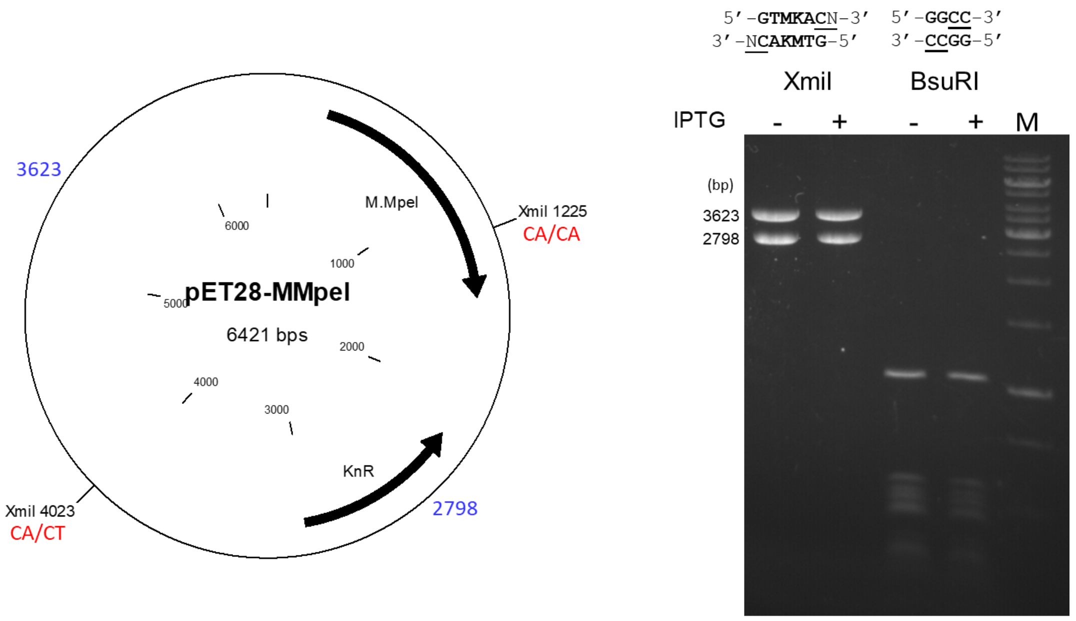

Supplementary Figure S1. Testing the sequence specificity of wild-type M.MpeI by digesting the plasmid pET28-MMpeI with XmiI and BsuRI. The map on the left shows the positions of the XmiI cleavage sites, the sizes of fragments obtained after complete digestion (in bp, blue), and the CN sites (in red) created by the 3′-C of the XmiI recognition sequence and the 3′-flanking nucleotide. There are 23 BsuRI sites in pET28-MMpeI. M, GeneRuler 1 kb DNA Ladder.

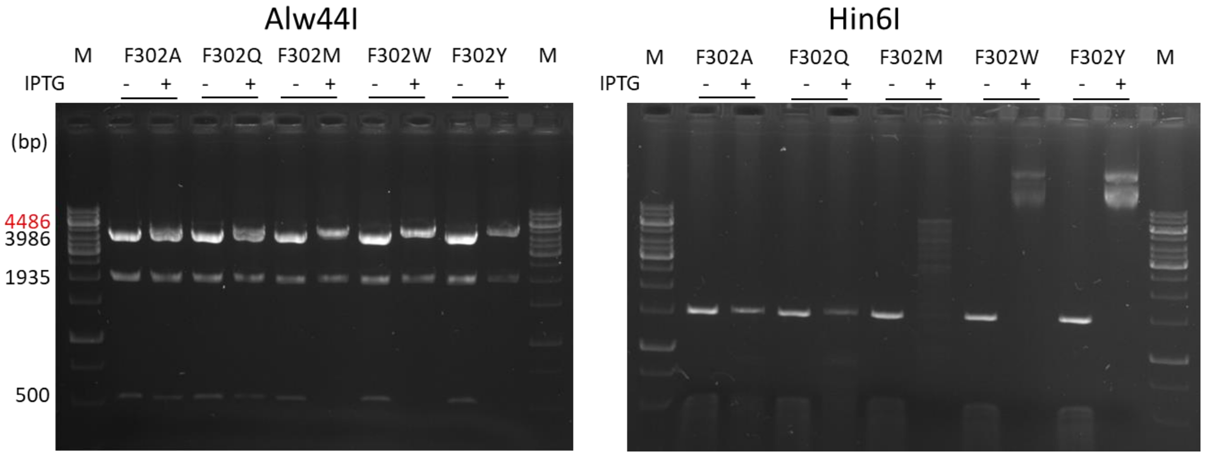

Supplementary Figure S2. Testing the sequence specificity of M.MpeI variants carrying substitutions of Phe302. Digestion of pET28-MMpeI(F302A/Q/M/W/Y) with Alw44I and Hin6I. For interpretation of the Alw44I fragment pattern, see Figure 1. There are 46 Hin6I sites in pET28-MMpeI. M, GeneRuler 1 kb DNA Ladder.

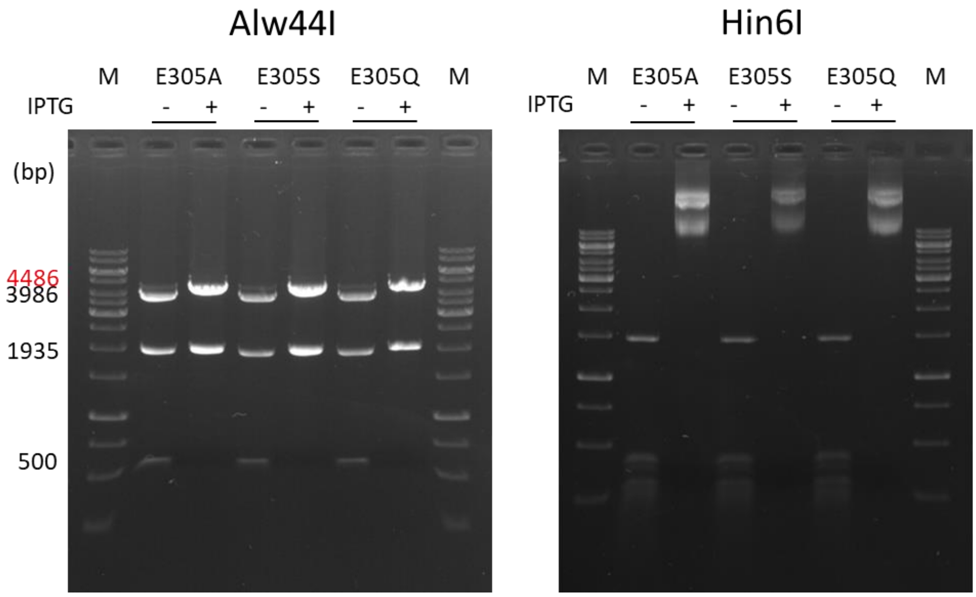

Supplementary Figure S3. Testing the sequence specificity of M.MpeI mutants carrying substitutions of Glu305. Digestion of pET28-MMpeI(E305A/S/Q) with Alw44I and Hin6I. Fragment size in red letters indicates protected fragment. For interpretation of the Alw44I fragment pattern, see Figure 1. M, GeneRuler 1 kb DNA Ladder.

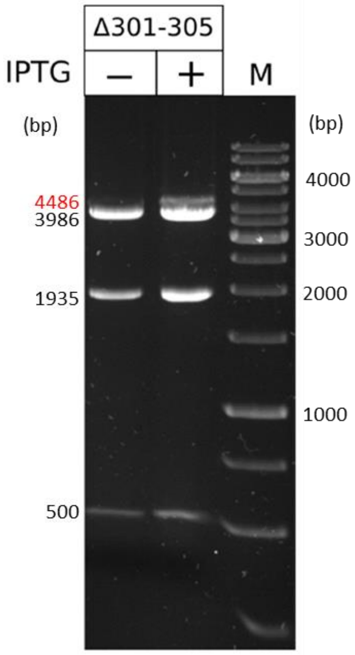

Supplementary Figure S4. Testing the sequence specificity of the M.MpeI variant carrying a deletion extending from Thr301 to Glu305. Digestion of pET28-MMpeI(Δ[T301-E305]) with Alw44I. Fragment size in red letters indicates protected fragment. For interpretation of the fragment pattern, see Figure 1. M, GeneRuler 1 kb DNA Ladder.

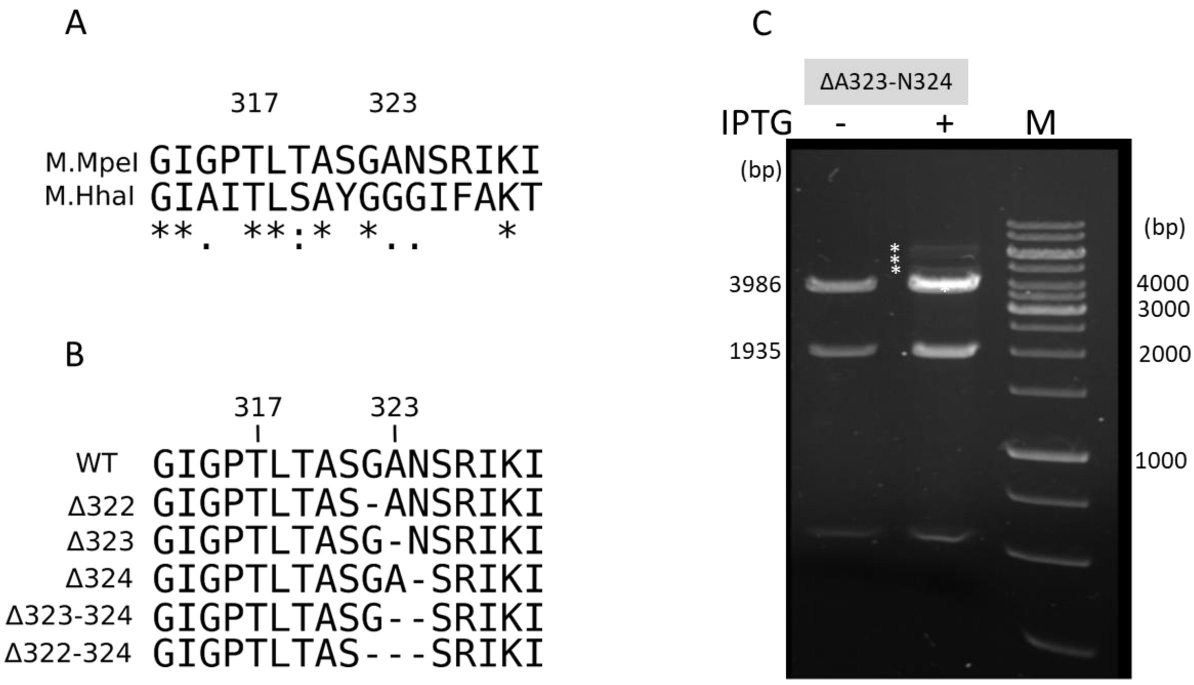

Supplementary Figure S5. Testing the sequence specificity of the M.MpeI variant from which the A323-N324 dipeptide has been deleted.

A, Partial amino acid sequence alignment of the Recognition Loops 2 of M.MpeI and M.HhaI.

B, Amino acid sequences of the M.MpeI deletion variants.

C, Digestion of pET28-MMpeIΔ[A323-N324] with Alw44I. Protected fragments (4480 bp, 5915 bp and 6415 bp) are indicated by asterisk. For interpretation of the fragment pattern, see Figure 1 (due to the Δ[A323-N324] deletion, the original 3986 bp fragment becomes shorter by 6 bp). M, GeneRuler 1 kb DNA Ladder.

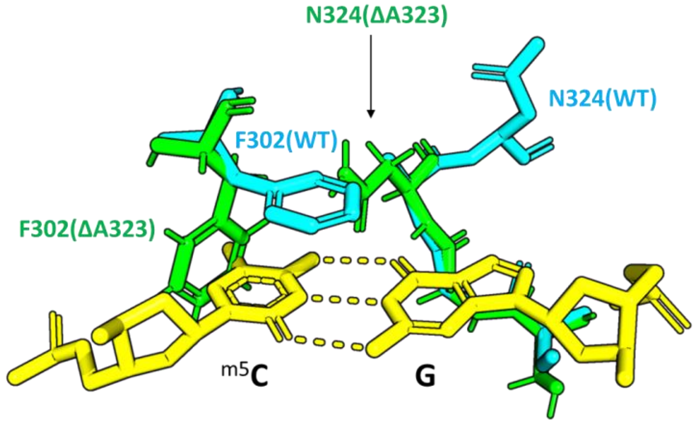

Supplementary Figure S6. Position of the Asn324 side chain in the wild-type and in the ΔA323 deletion mutant M.MpeI. Superimposition of the X-ray structure of the wild-type enzyme and of the predicted structure of the mutant enzyme. Yellow, DNA; cyan, wild-type; green, ΔA323 variant.

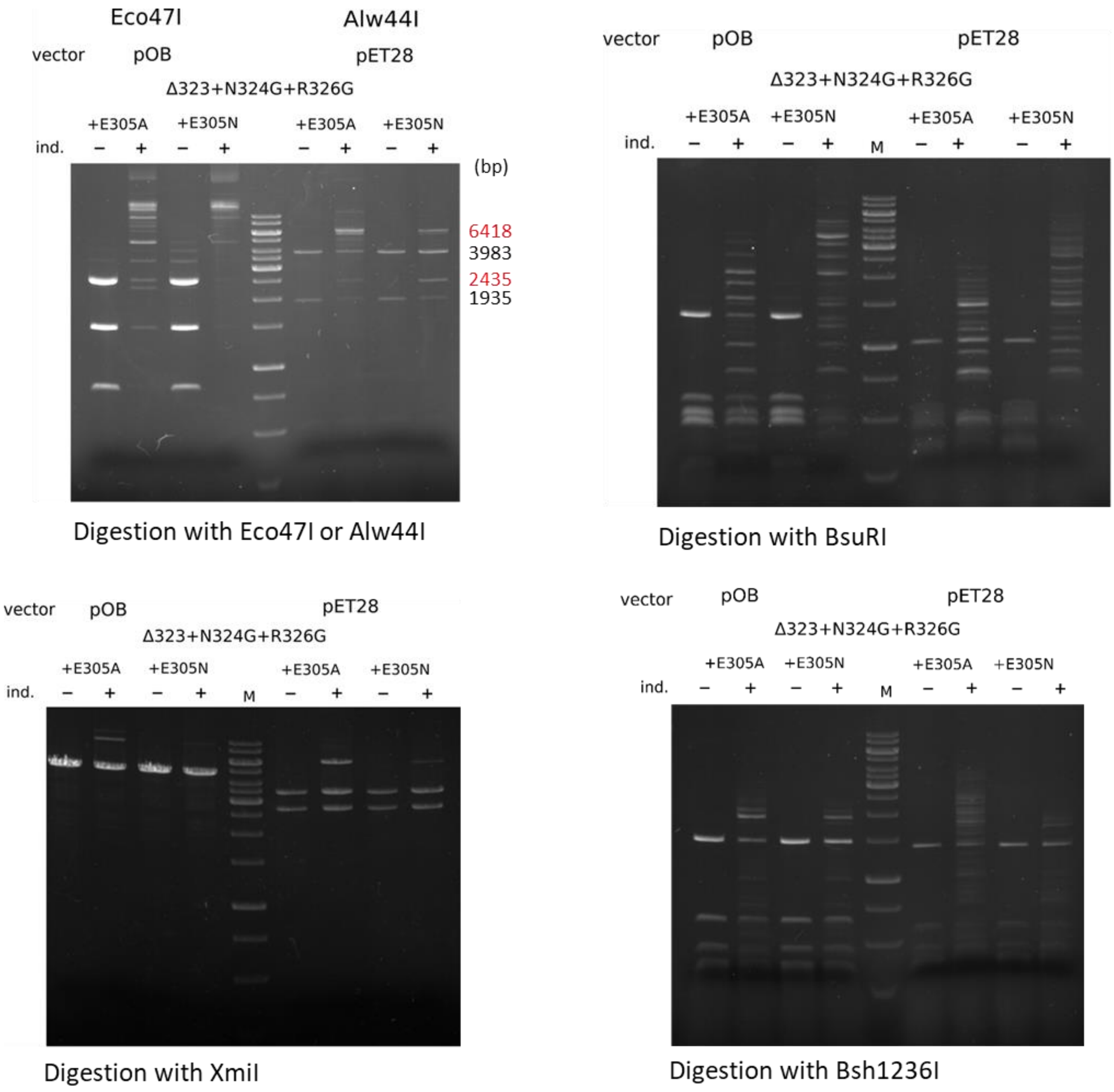

Supplementary Figure S7. Testing the methylation specificity of M.MpeI(ΔA323+N324G+R326G+E305A) and M.MpeI(ΔA323+N324G+R326G+E305N) expressed from pOK-BAD or pET28 vector plasmids. The plasmids encoding the respective variants were digested with the indicated enzymes. Plasmid preparations marked with + were isolated from cultures induced with arabinose or IPTG. Sizes of fragments protected against Alw44I digestion are shown in red. For interpretation of the fragment pattern, see Figure 1 (Alw44I), Figure 6 (Eco47I) and Supplementary Figure S1 (XmiI). M, GeneRuler 1 kb DNA Ladder.

A

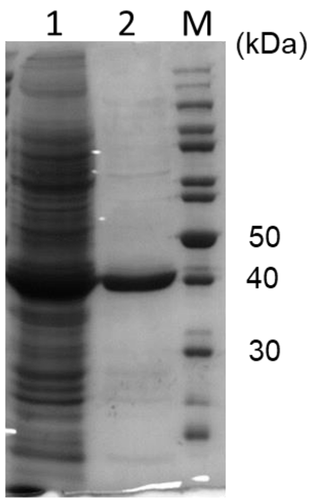

B

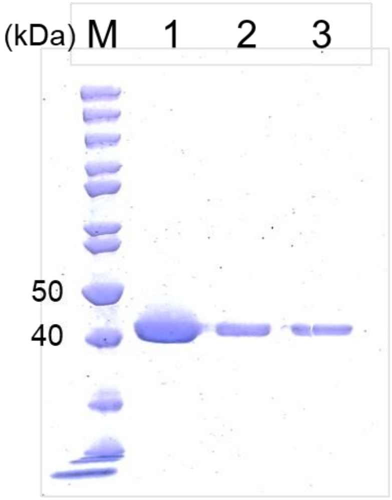

C

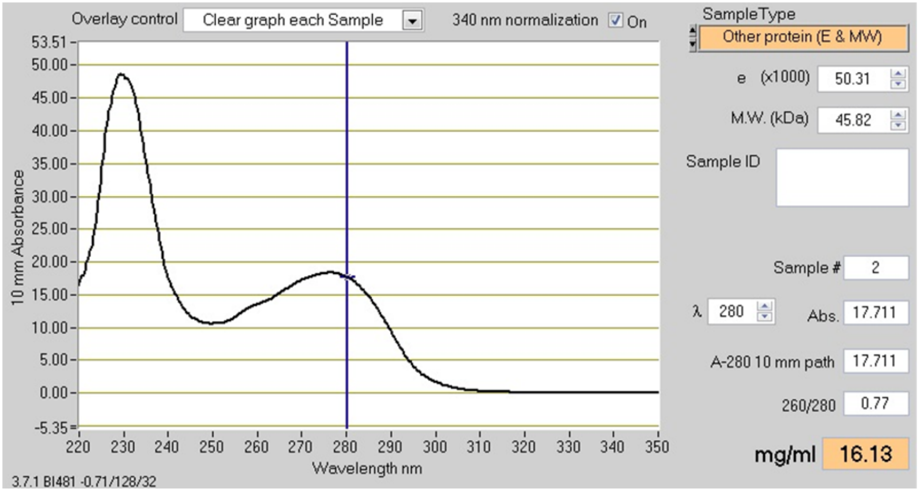

Supplementary Figure S8. Analysis of M.MpeI(ΔA323+N324G+R326G+E305N) purified by affinity chromatography.

A, SDS-polyacrylamide gel electrophoresis of the enzyme preparation obtained with His-Spin Protein Miniprep column (Zymo Research). Lane 1, Supernatant prepared from IPTG-induced *E. coli* ScarabXpress T7 *lac* cells carrying pET28-MMpeI(ΔA323+N324G+R326G+E305N); Lane 2, purified enzyme. M, Page Ruler Unstained Protein Ladder (Thermo Fisher Scientific)

B, SDS-polyacrylamide gel electrophoresis of the enzyme preparation obtained with HIS-Select Nickel Affinity Gel (Sigma-Aldrich) chromatography. Lane 1, 2 µl of 10-fold diluted HIS-SELECT purified enzyme; Lane 2, 2 µl of 66-fold diluted HIS-SELECT purified enzyme; Lane 3, 2 µl of 5-fold diluted preparation obtained using His-Spin Protein Miniprep column.

C, Determination of the concentration of M.MpeI(ΔA323+N324G+R326G+E305N) purified by HIS-Select Nickel Affinity Gel. UV absorption obtained by Nanodrop Spectrophotometer (Thermo Fisher Scientific).

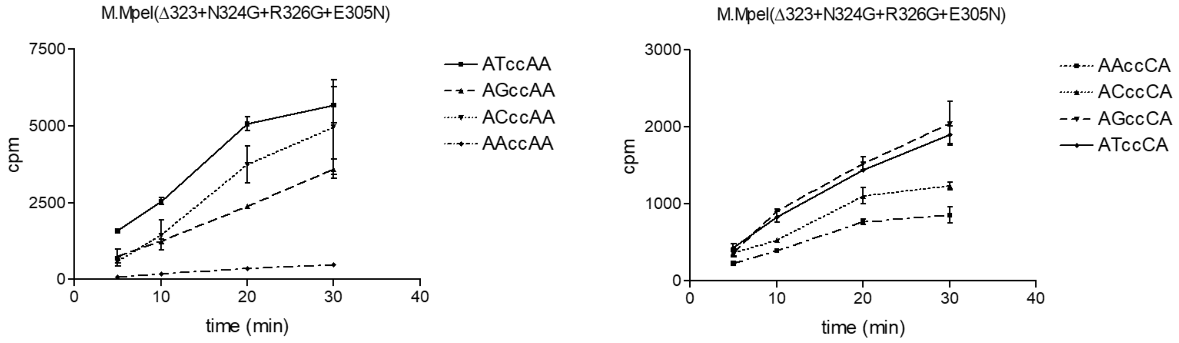

Supplementary Figure S9. Time course of methyl transfer by MpeI(ΔA323+N324G+R326G+E305N) into CC sites in different sequence contexts. Sequences of the double-stranded oligonucleotides differed in the two nucleotides preceding and following the cc site (Supplementary Table S6). Average values of three independent experiments. Error bars: standard error of the mean.

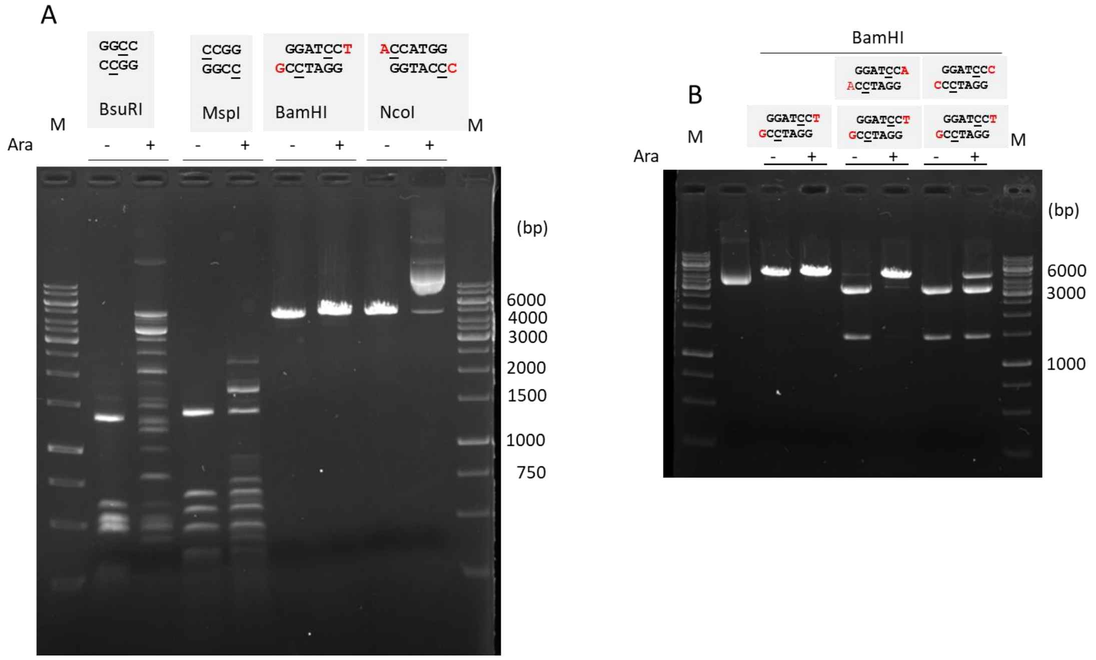

Supplementary Figure S10. Effects of the flanking bases on the CC-specific activity of M.MpeI(ΔA323+N324G+R326G+E305N) *in vivo*. The plasmids were purified from uninduced and arabinose-induced cultures and digested with the indicated enzymes whose cleavage is blocked by C5-methylation of the underlined cytosines.

A, pOB-MMpeI(ΔA323+N324G+R326G+E305N) digested with BsuRI, MspI, BamHI and NcoI. The plasmid contains 13 BsuRI, 19 MspI, 1 BamHI and 1 NcoI sites. Relevant nucleotides bordering the BamHI and the NcoI sites are shown in red.

B, Plasmids pOB-MMpeI(ΔA323+N324G+R326G+E305N), pOB-MMpeI(ΔA323+N324G+R326G+E305N)-AK946 and pOB-MMpeI(ΔA323+N324G+R326G+E305N)-948 digested with BamHI. The plasmids contain one or two BamHI sites with different 3’ flanking nucleotides (in red) as shown above the gel.

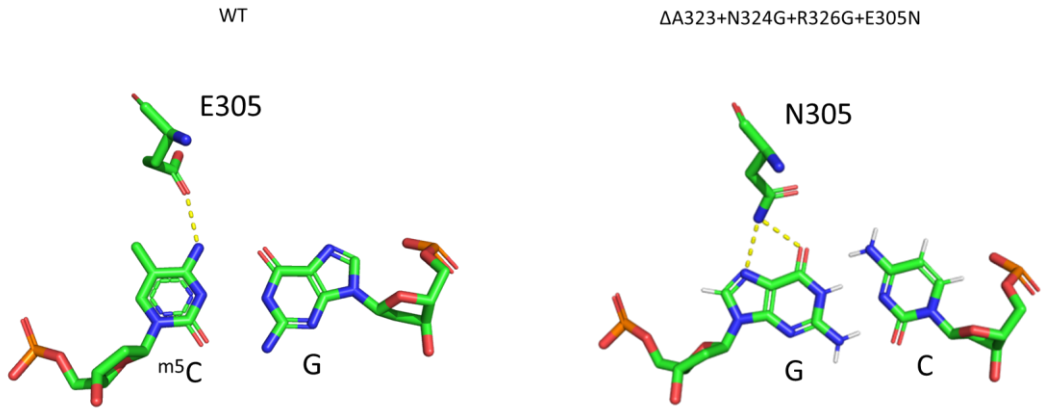

Supplementary Figure S11. PyMOL rendering of the relevant parts of the structures of the specific enzyme-DNA complexes formed by the wild-type and the quadruple mutant M.MpeI. The wild-type structure was determined by X-ray crystallography (Wojciechowski, 2013). Structure of the mutant enzyme was generated from the wild-type structure using the I-Tasser platform (4).

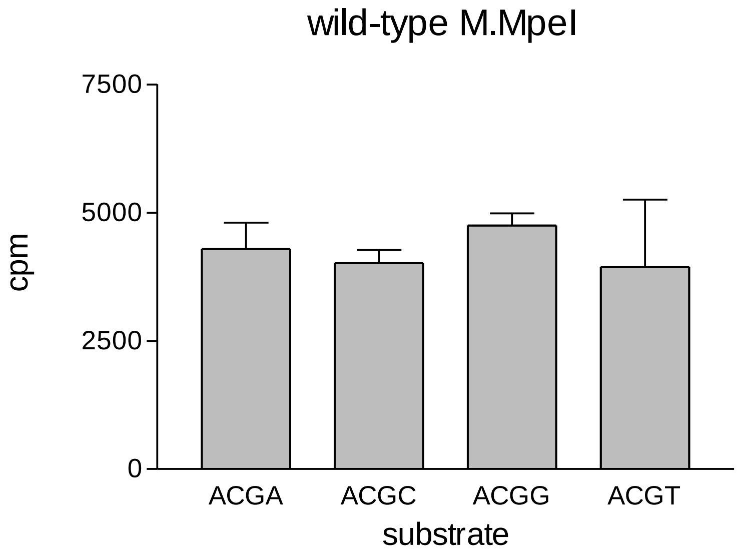

Supplementary Figure S12. Effects of the 3'-flanking nucleotides on the activity of wild-type M.MpeI. *In vitro* MTase reactions containing 10 nM purified wild-type M.MpeI and the double stranded oligonucleotides AK702-AK703 (ACGA), AK1008-AK1009 (ACGC), AK1010-AK1011 (ACGG) and AK1012-AK1013 (ACGT) (Supplementary Tables S2 and S6). The reaction conditions were the same as described above for the *in vitro* radioactive assay except that 10 min reactions were used. Average values of three measurements. Error bars: standard error of the mean.

REFERENCES

1. Roberts, R.J., Vincze, T., Posfai, J. and Macelis, D. (2022) REBASE: a database for DNA restriction and modification: enzymes, genes and genomes. *Nucleic Acids Res.*, **51**, D629-D630.

2. Tímár, E., Venetianer, P. and Kiss, A. (2008) In vivo DNA protection by relaxed-specificity SinI DNA methyltransferase variants. *J. Bacteriol.*, **190**, 8003-8008.
3. Wojciechowski, M., Czapinska, H. and Bochtler, M. (2013) CpG underrepresentation and the bacterial CpG-specific DNA methyltransferase M.MpeI. *Proc. Natl. Acad. Sci. USA*, **110**, 105-110.
4. Roy, A., Kucukural, A. and Zhang, Y. (2010) I-TASSER: a unified platform for automated protein structure and function prediction. *Nat. Protoc.*, **5**, 725-738.
